# Supplementary material for: Spectral Assignment in the [3 + 2] Cycloadditions of Methyl (2E)-3-(Acridin-4-yl)-prop-2-enoate and 4-[(E)-2-Phenylethenyl]acridin with Unstable Nitrile N-Oxides
Source: Molecules. 2024 Jun 9;29(12):2756. doi: 10.3390/molecules29122756 (PMC11206844; doi:10.3390/molecules29122756)
Supplement: Supplementary file 1 [file molecules-29-02756-s001.zip › molecules-3031626-supplementary.pdf]

Supplementary Information

Spectral Assignment in the [3+2] Cycloadditions of Methyl 3-(Acridin-4-yl)-Acrylate and 4-[(*E*)-2-Phenylethenyl]acridin with Unstable Nitrile Oxides

Lucia Ungvarská Maľučká[a,b] and Mária Vilková[a*]

[a] *Pavol Jozef Šafárik University, Faculty of Science, Institute of Chemistry, Moyzesova 11, 040 01 Košice, Slovakia*
[b] *The University of veterinary medicine and pharmacy in Košice, Department of Chemistry, Biochemistry and Biophysics, Komenského 73, 041 81 Košice, Slovakia*

Corresponding author: maria.vilkova@upjs.sk

Keywords: [3+2] cycloadditions; regioselectivity explanation; structure elucidation; geometric isomers resolution; relative configuration determination; 1D and 2D NMR spectroscopy

Content

1 NMR spectra of synthesised derivatives 2
1.1 Methyl 4-(acridin-4-yl)-3-phenyl-4,5-dihydro-1,2-oxazole-5-carboxylate (5b) 2
1.2 Methyl 5-(acridin-4-yl)-3-(4-methoxyphenyl)-4,5-dihydro-1,2-oxazole-4-carboxylate (6a) 6
1.3 Methyl 5-(acridin-4-yl)-3-phenyl-4,5-dihydro-1,2-oxazole-4-carboxylate (6b) 7
1.4 Methyl 5-(acridin-4-yl)-3-(3-nitrophenyl)-4,5-dihydro-1,2-oxazole-4-carboxylate (6d) 10
1.5 Methyl 5-(acridin-4-yl)-3-(4-nitrophenyl)-4,5-dihydro-1,2-oxazole-4-carboxylate (6e) 11
1.6 4-(3,5-Diphenyl-4,5-dihydro-1,2-oxazol-4-yl)acridine (7b) 12
1.7 4-[3-(3-Nitrophenyl)-5-phenyl-4,5-dihydro-1,2-oxazol-4-yl]acridine (7d) and 4-[3-(3-nitrophenyl)-4-phenyl-4,5-dihydro-1,2-oxazole-5-yl]acridine (8d) 15
1.8 4-[3-(4-Nitrophenyl)-5-phenyl-4,5-dihydro-1,2-oxazol-4-yl]acridine (7e) and 4-[3-(4-nitrophenyl)-4-phenyl-4,5-dihydro-1,2-oxazol-5-yl]acridine (8e) 16
1.9 4-[3-(4-Methoxyphenyl)-4-phenyl-4,5-dihydro-1,2-oxazol-5-yl]acridine (8a) 17
1.10 4-(3,4-Diphenyl-4,5-dihydro-1,2-oxazol-5-yl)acridine (8b) 18
1.11 4-[3-(4-Bromophenyl)-4-phenyl-4,5-dihydro-1,2-oxazol-5-yl]acridine (8c) 21
1.12 5-(Acridin-4-yl)-3-(4-methoxyphenyl)-4,5-dihydro-1,2-oxazole-4-carboxylic acid (9a) 22
1.13 5-(Acridin-4-yl)-3-phenyl-4,5-dihydro-1,2-oxazole-4-carboxylic acid (9b) 23
1.14 5-(Acridin-4-yl)-3-(3-nitrophenyl)-4,5-dihydro-1,2-oxazole-4-carboxylic acid (9d) and 4-[3-(3-nitrophenyl)-4,5-dihydro-1,2-oxazol-5-yl]acridine (11d) 26
1.15 5-(Acridin-4-yl)-3-(4-nitrophenyl)-4,5-dihydro-1,2-oxazole-4-carboxylic acid (9e) 27
1.16 (4*Z*)-4-[(Acridin-4-yl)methylidene)]-3-(4-nitrophenyl)-4,5-dihydro-1,2-oxazol-5-one (*Z*-10e) 28
1.17 4-[3-(4-Methoxyphenyl)-4,5-dihydro-1,2-oxazol-5-yl]acridine (11a) 32
1.18 4-(3-Phenyl-4,5-dihydro-1,2-oxazol-5-yl)acridine (11b) 33
1.19 4-[3-(4-Nitrophenyl)-4,5-dihydro-1,2-oxazol-5-yl]acridine (11e) 34

1 NMR spectra of synthesised derivatives

1.1 Methyl 4-(acridin-4-yl)-3-phenyl-4,5-dihydro-1,2-oxazole-5-carboxylate (5b)

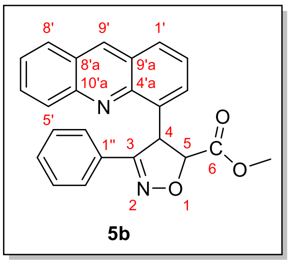

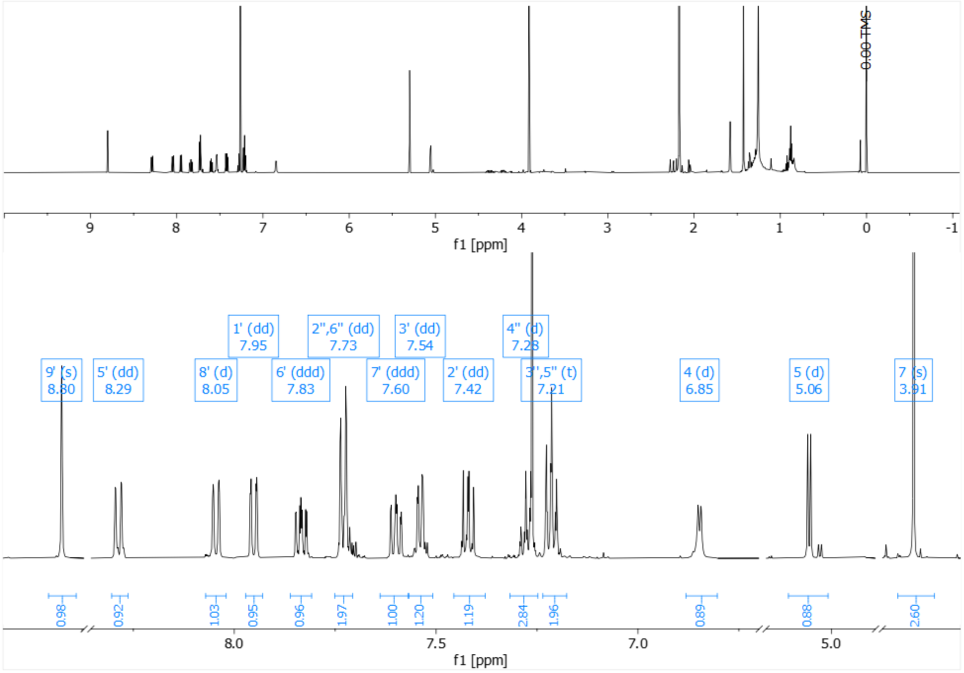

Figure S1. ^{1}H (600 MHz, CDCl$_3$) NMR spectrum of derivative **5b**.

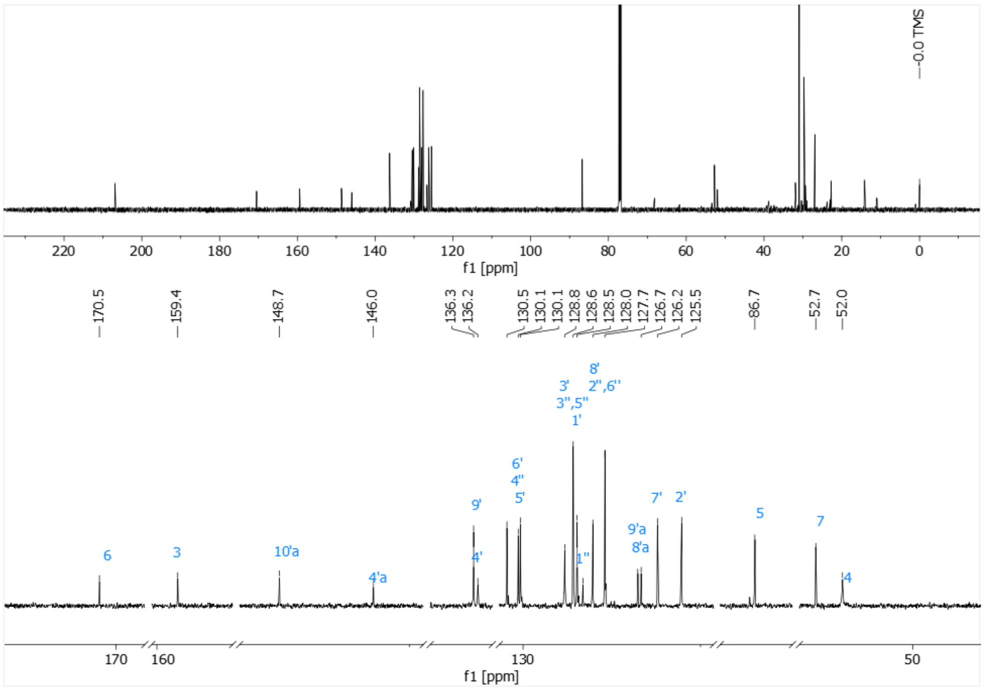

Figure S2. ^{13}C NMR (150 MHz, CDCl$_3$) NMR spectrum of derivative **5b**.

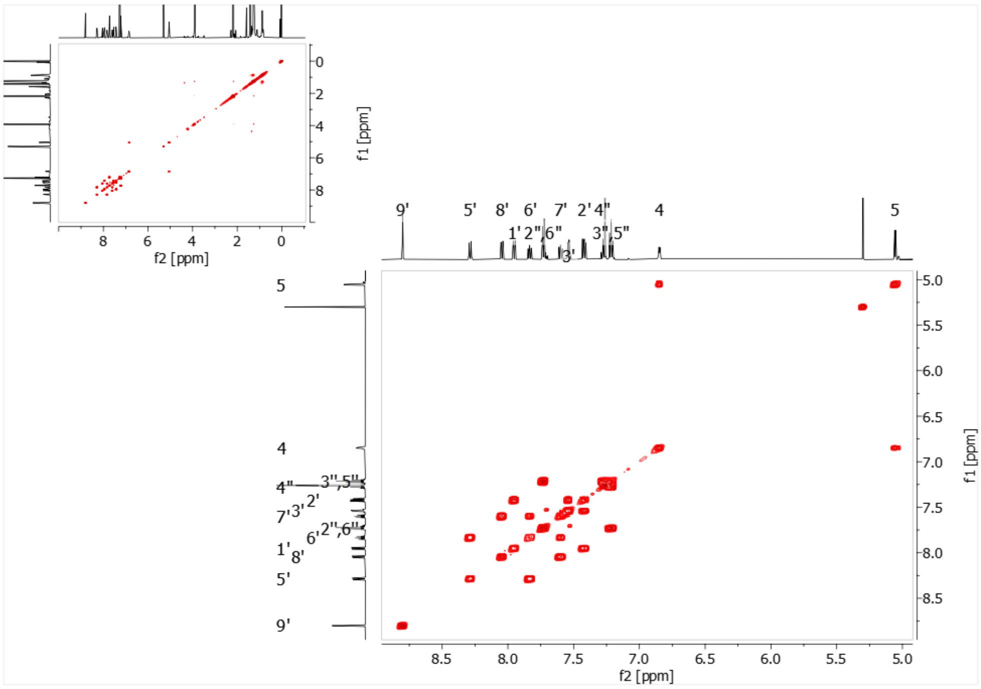

Figure S3. COSY ($CDCl_3$) NMR spectrum of derivative **5b**.

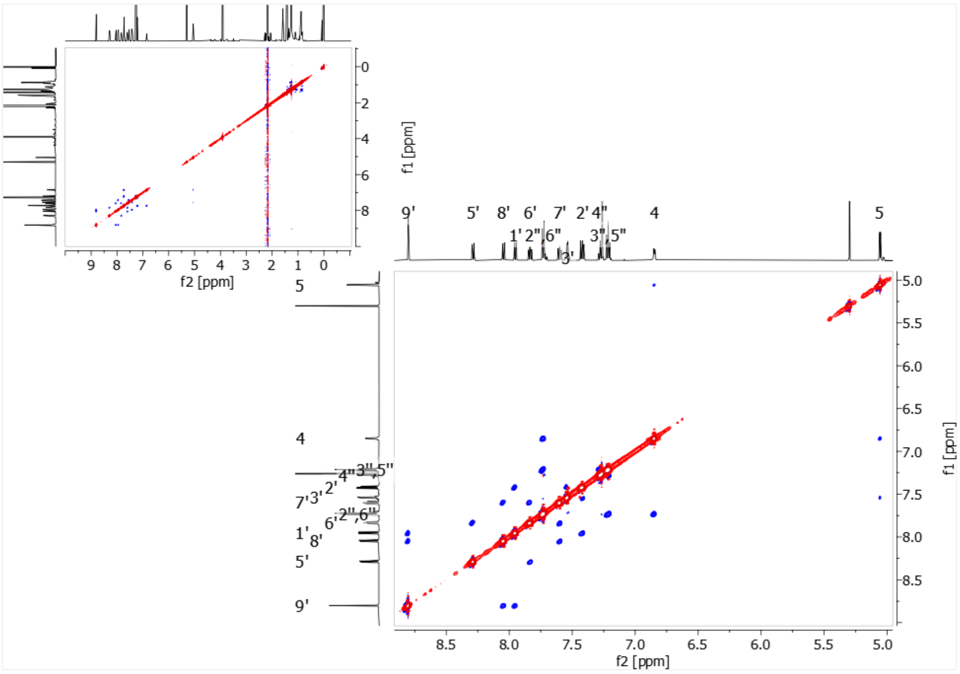

Figure S4. NOESY ($CDCl_3$) NMR spectrum of derivative **5b**.

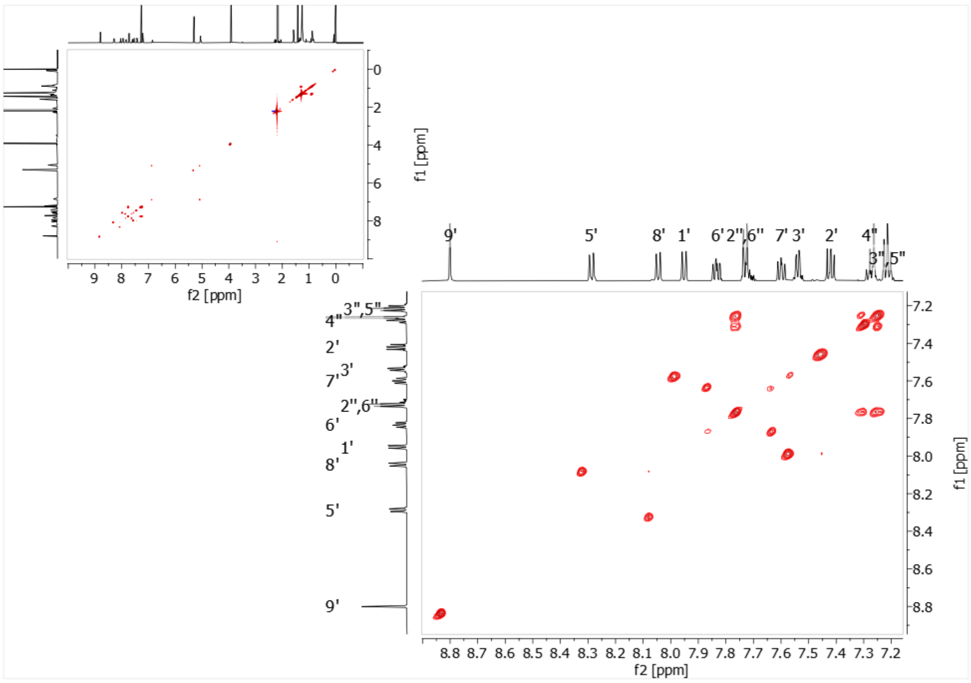

Figure S5. TOCSY ($CDCl_3$) NMR spectrum of derivative **5b**.

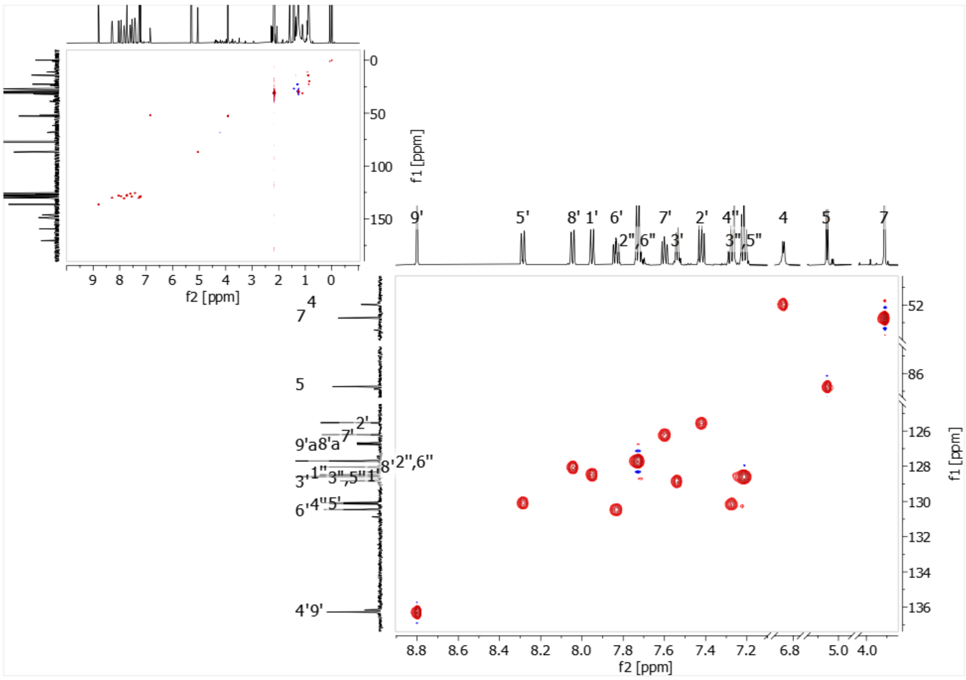

Figure S6. $^{1}H,^{13}C$-HSQC ($CDCl_3$) NMR spectrum of derivative **5b**.

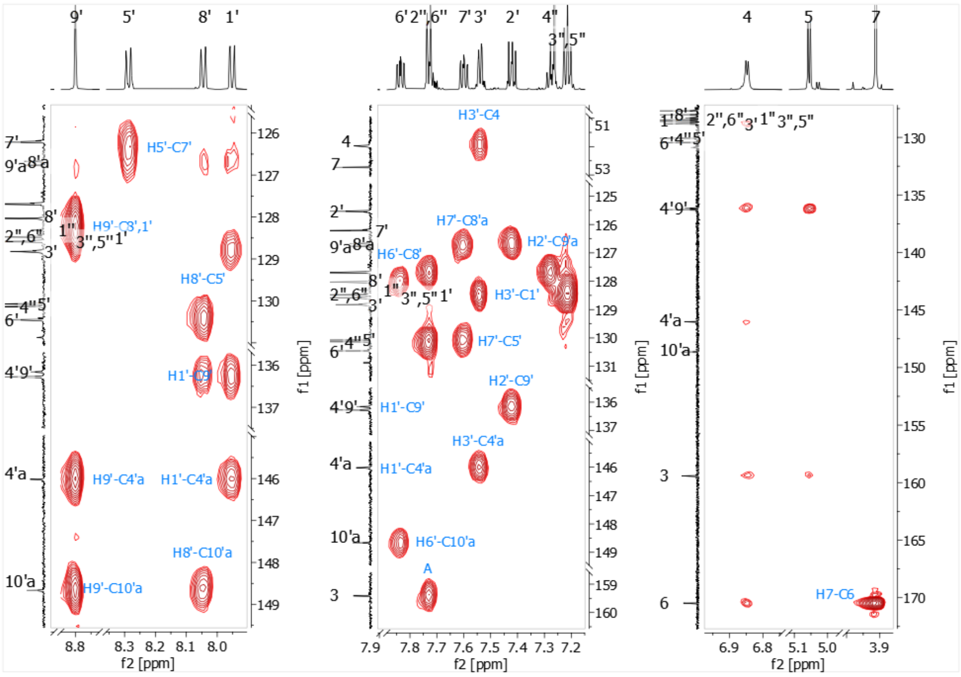

Figure S7. $^{1}H,^{13}C$-HMBC ($CDCl_3$) NMR spectrum of derivative **5b**.

1.2 Methyl 5-(acridin-4-yl)-3-(4-methoxyphenyl)-4,5-dihydro-1,2-oxazole-4-carboxylate (6a)

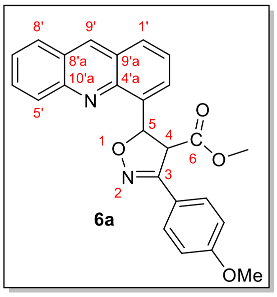

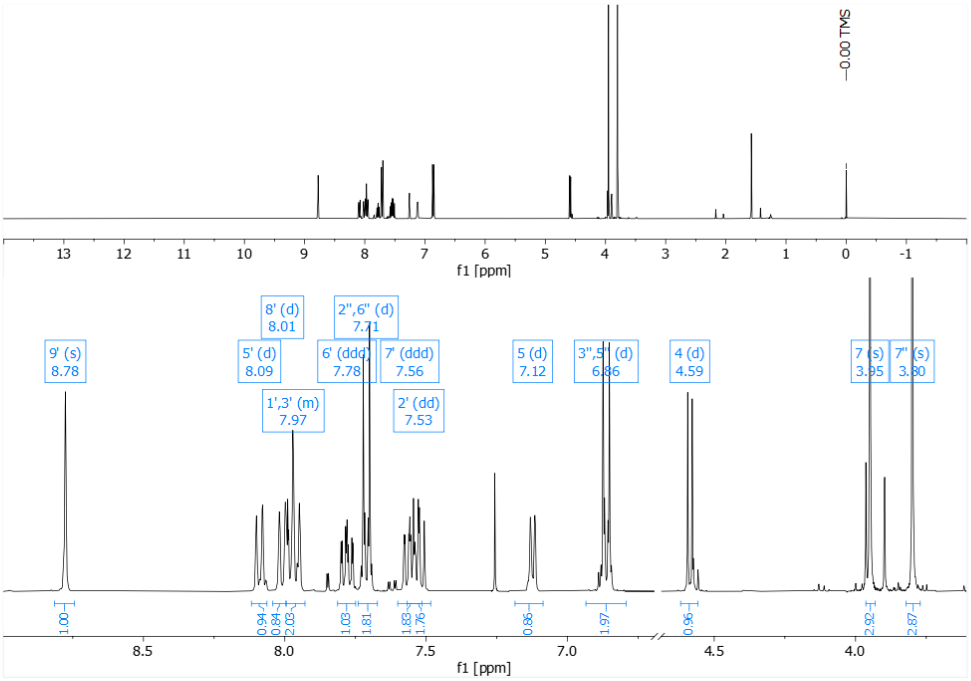

Figure S8. ^{1}H (400 MHz, $CDCl_3$) NMR spectrum of derivative **6a**.

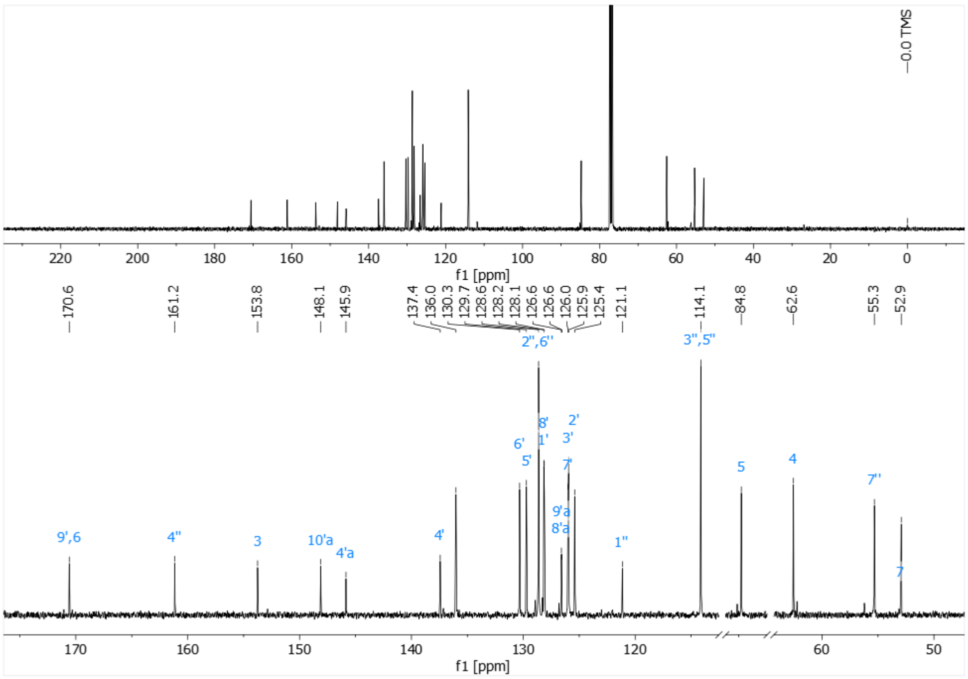

Figure S9. ^{13}C (100 MHz, $CDCl_3$) NMR spectrum of derivative **6a**.

1.3 Methyl 5-(acridin-4-yl)-3-phenyl-4,5-dihydro-1,2-oxazole-4-carboxylate (6b)

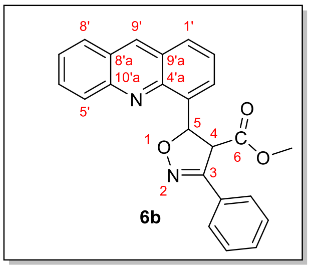

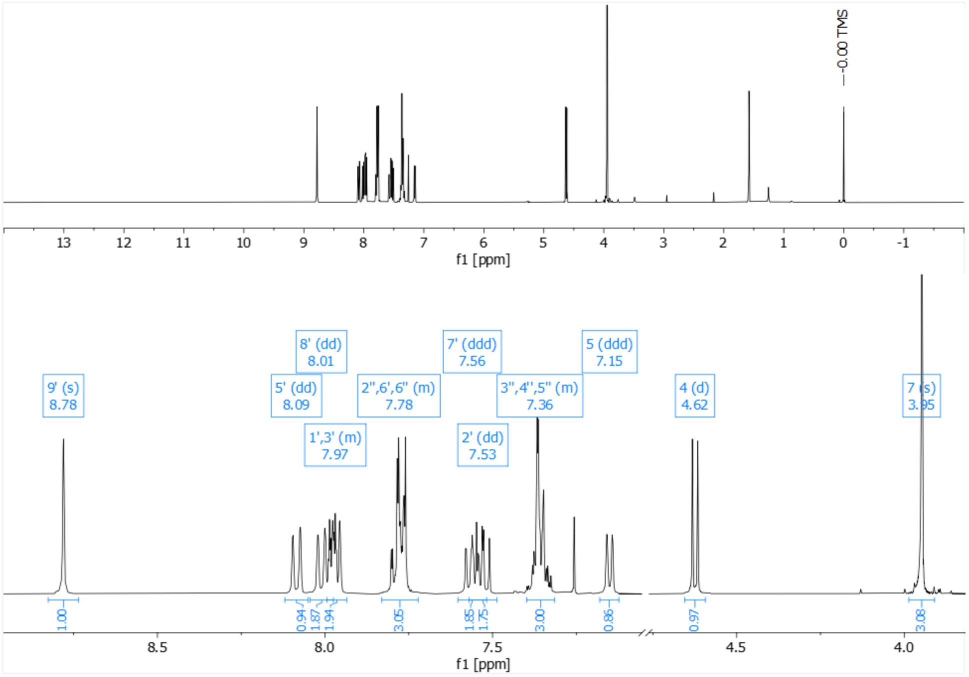

Figure S10. ^{1}H (400 MHz, $CDCl_3$) NMR spectrum of derivative **6b**.

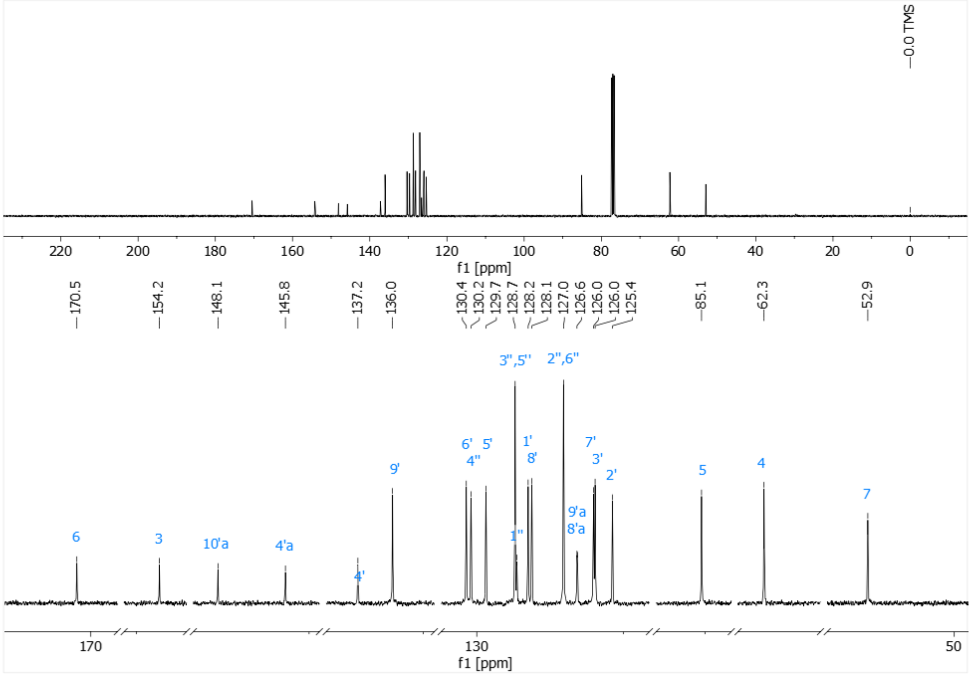

Figure S11. ^{13}C (100 MHz, $CDCl_3$) NMR spectrum of derivative **6b**.

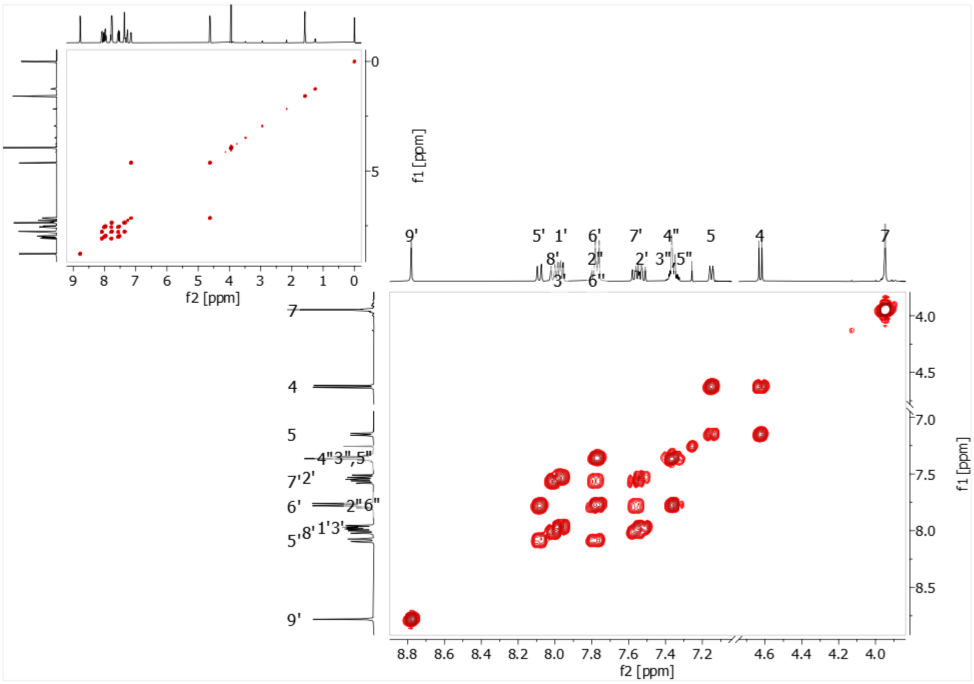

Figure S12. COSY ($CDCl_3$) NMR spectrum of derivative **6b**.

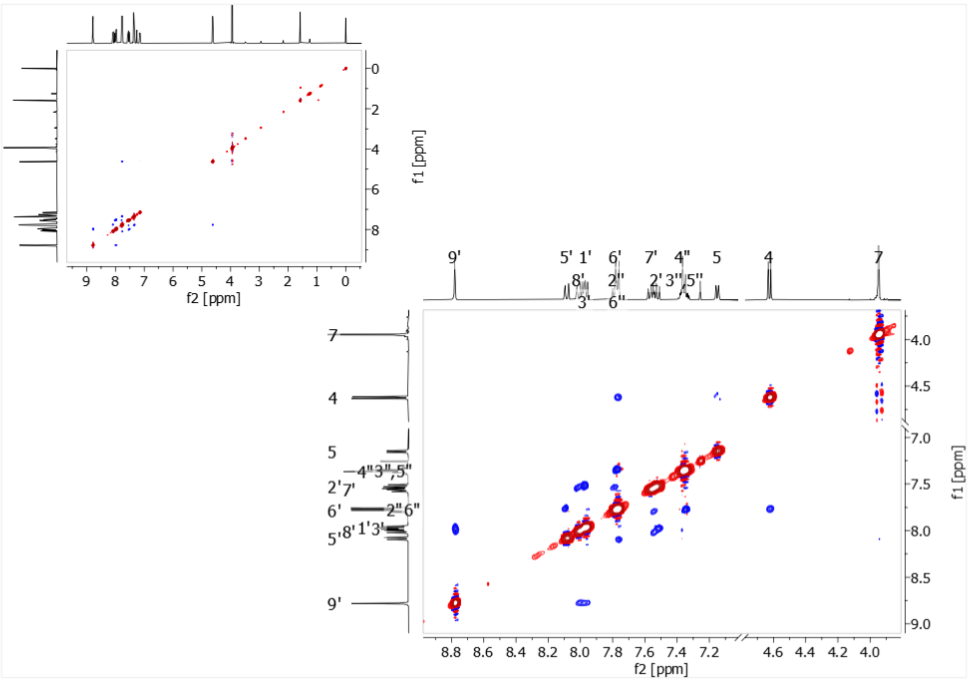

Figure S13. NOESY ($CDCl_3$) NMR spectrum of derivative **6b**.

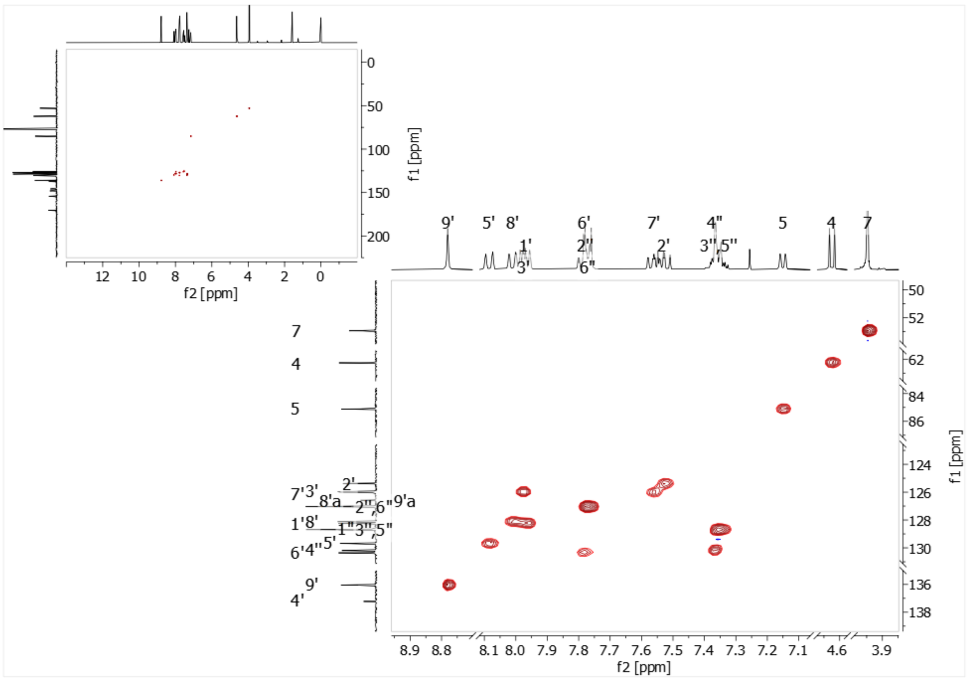

Figure S14. $^{1}H,^{13}C$-HSQC ($CDCl_3$) NMR spectrum of derivative **6b**.

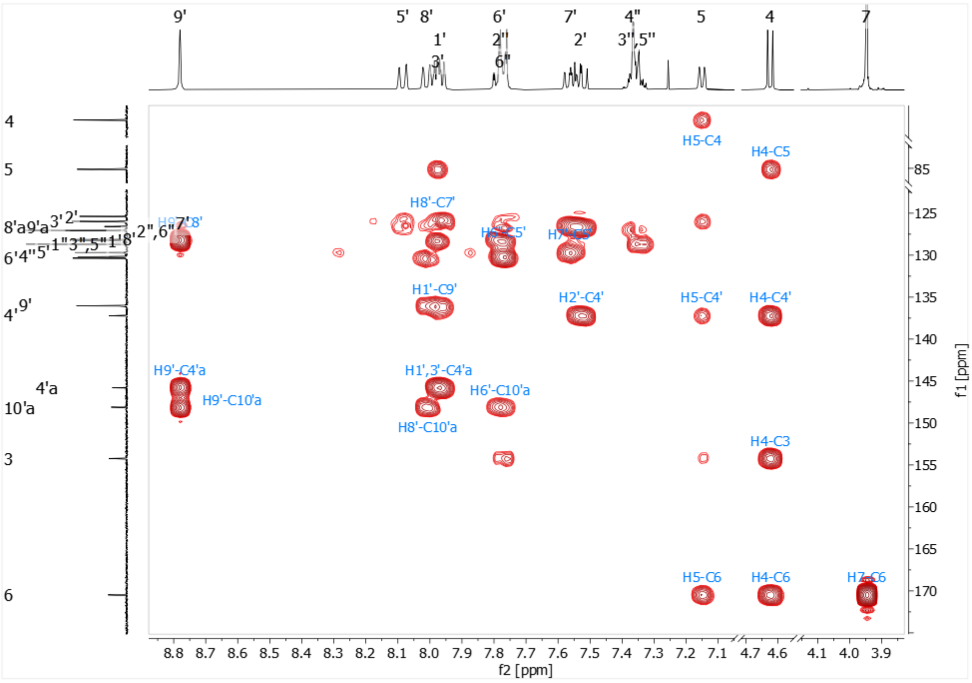

Figure S15. $^{1}H,^{13}C$-HMBC ($CDCl_3$) NMR spectrum of derivative **6b**.

1.4 Methyl 5-(acridin-4-yl)-3-(3-nitrophenyl)-4,5-dihydro-1,2-oxazole-4-carboxylate (6d)

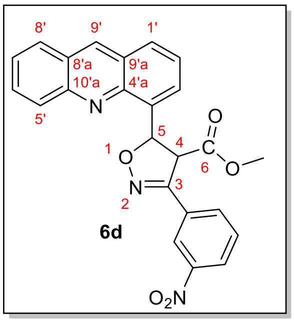

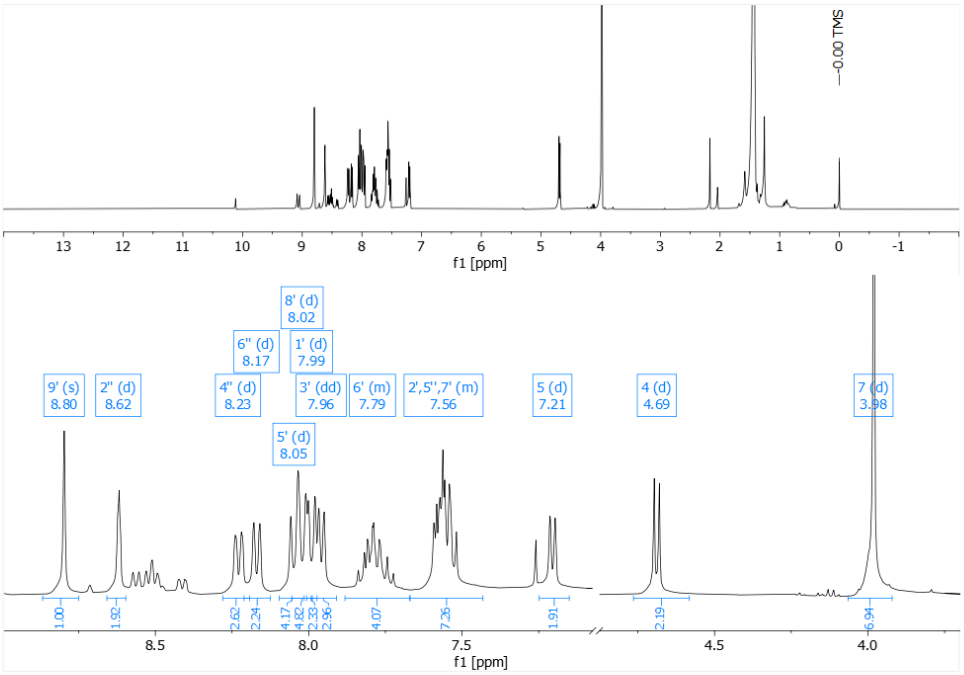

Figure S16. ¹H (400 MHz, CDCl₃) NMR spectrum of derivative **6d**.

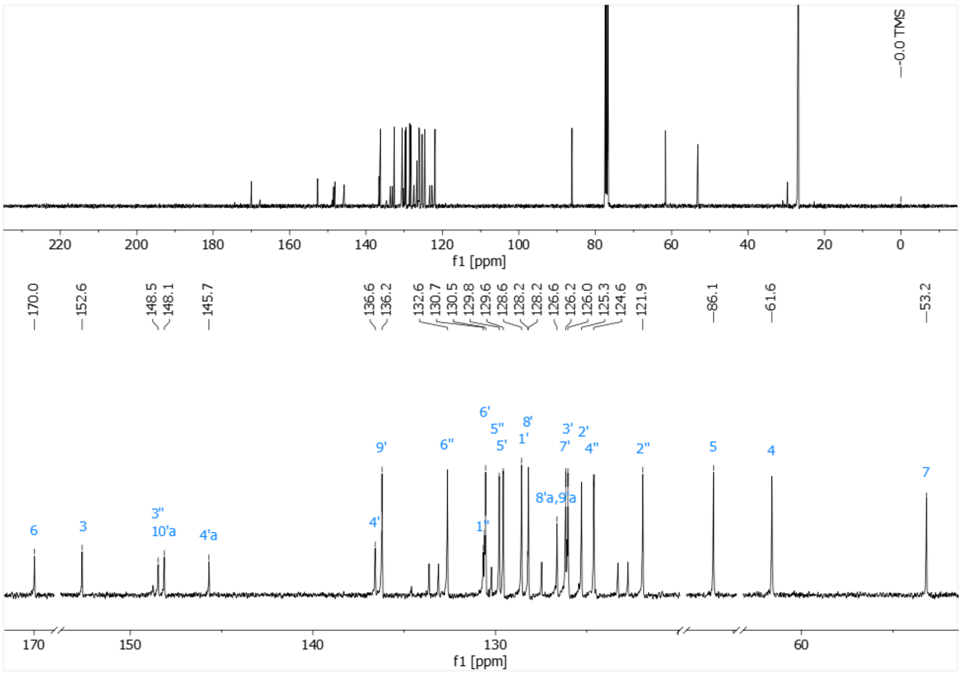

Figure S17. ¹³C (100 MHz, CDCl₃) NMR spectrum of derivative **6d**.

1.5 Methyl 5-(acridin-4-yl)-3-(4-nitrophenyl)-4,5-dihydro-1,2-oxazole-4-carboxylate (6e)

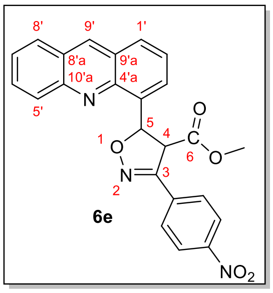

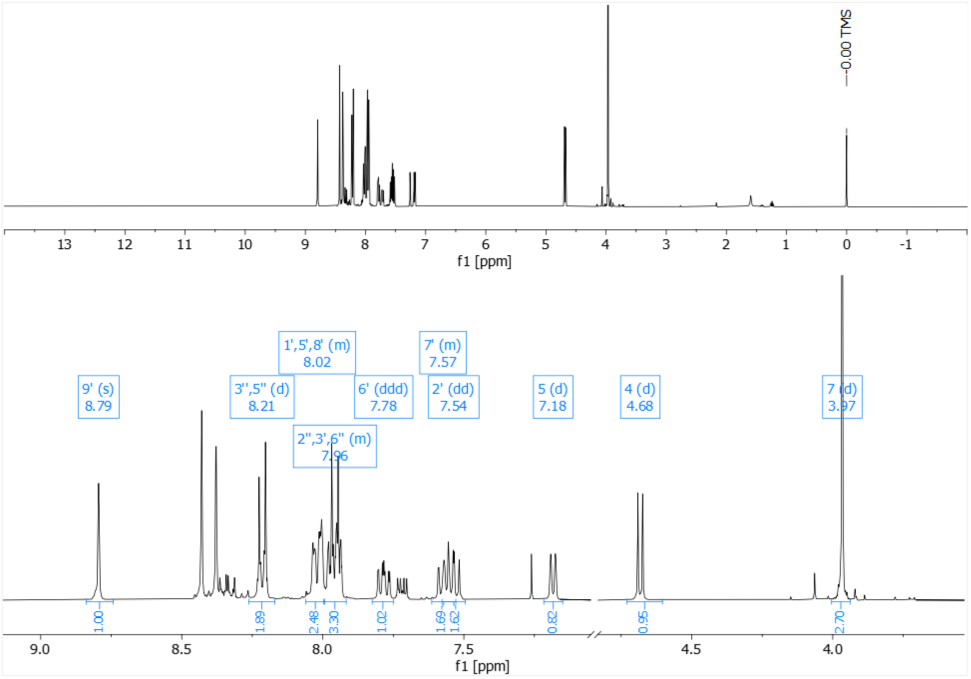

Figure S18. ^{1}H (400 MHz, $CDCl_3$) NMR spectrum of derivative **6e**.

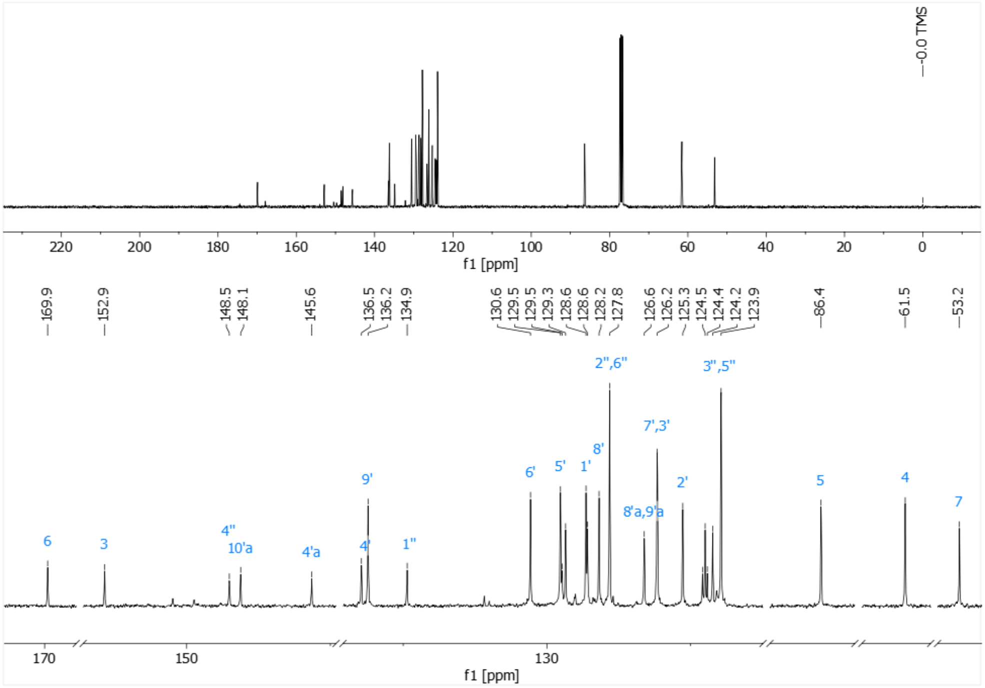

Figure S19. ^{13}C (100 MHz, $CDCl_3$) NMR spectrum of derivative **6e**.

1.6 4-(3,5-Diphenyl-4,5-dihydro-1,2-oxazol-4-yl)acridine (7b)

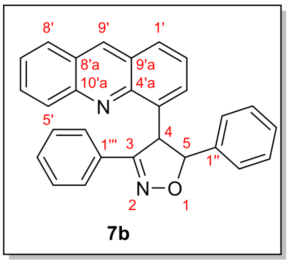

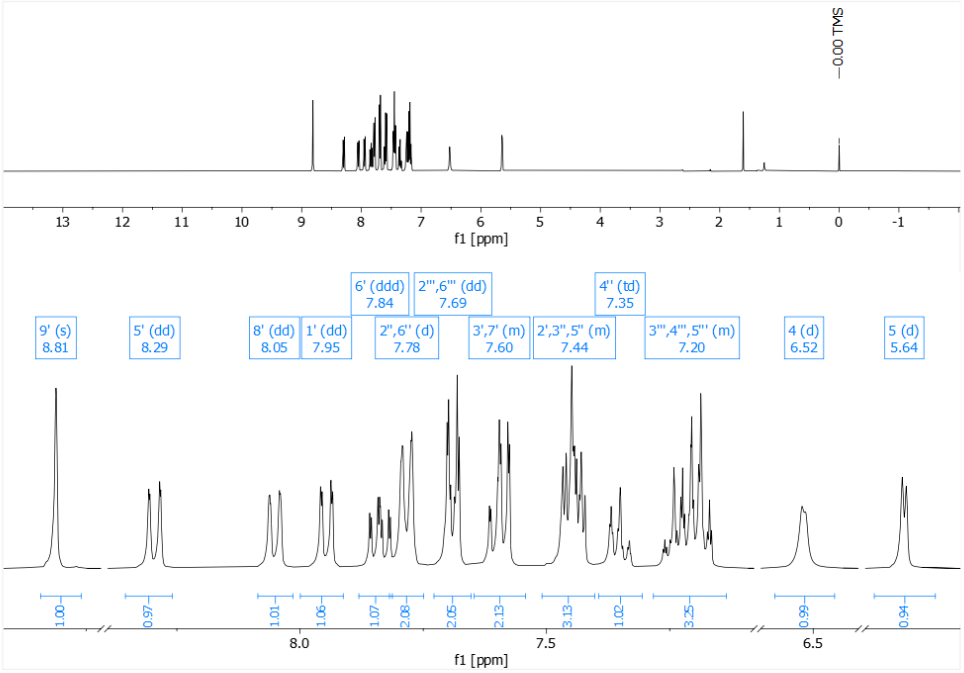

Figure S20. ¹H (400 MHz, CDCl₃) NMR spectrum of derivative **7b**.

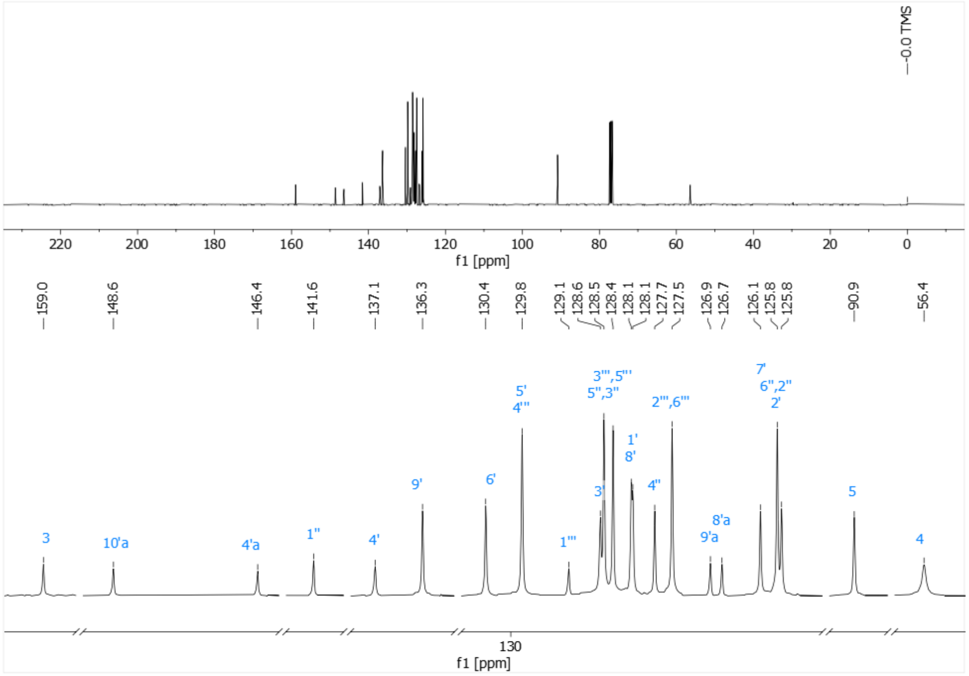

Figure S21. ^{13}C (100 MHz, CDCl$_3$) NMR spectrum of derivative **7b**.

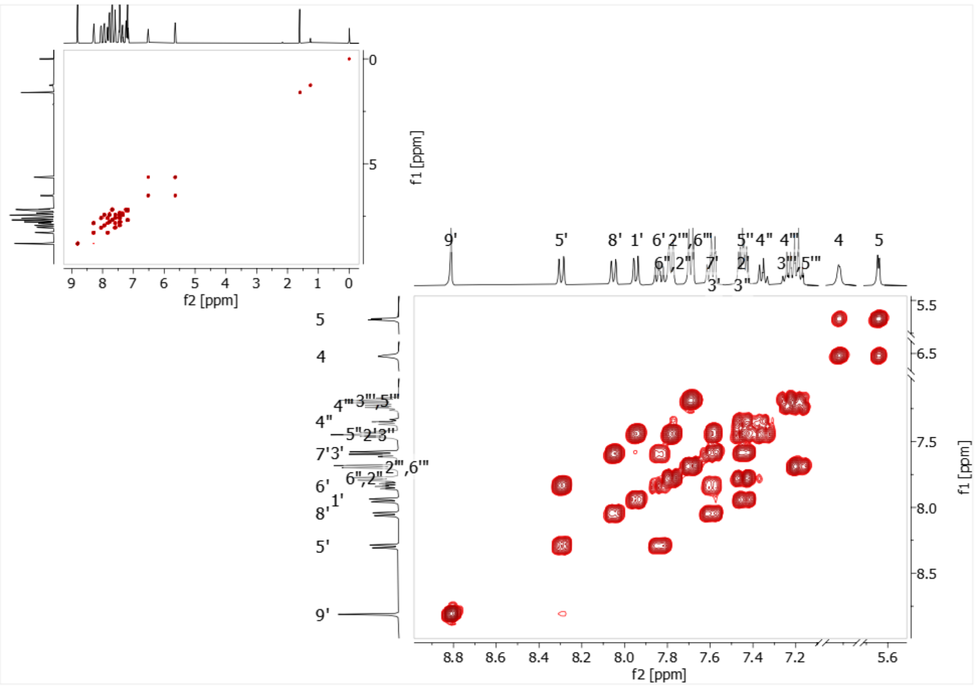

Figure S22. COSY ($CDCl_3$) NMR spectrum of derivative **7b**.

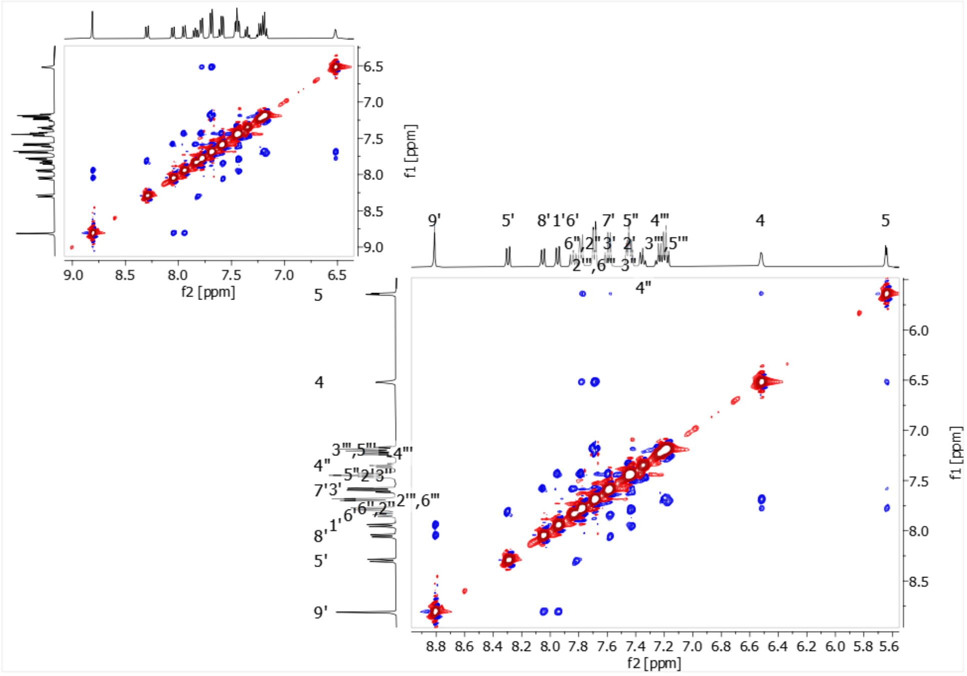

Figure S23. NOESY ($CDCl_3$) NMR spectrum of derivative **7b**.

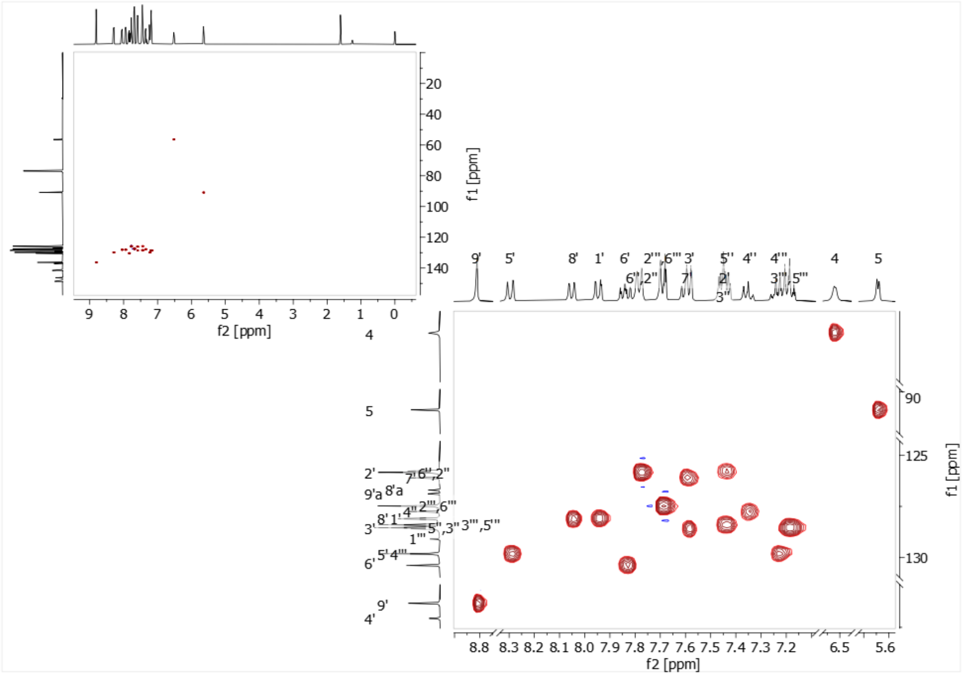

Figure S24. ^{1}H,^{13}C-HSQC ($CDCl_3$) NMR spectrum of derivative **7b**.

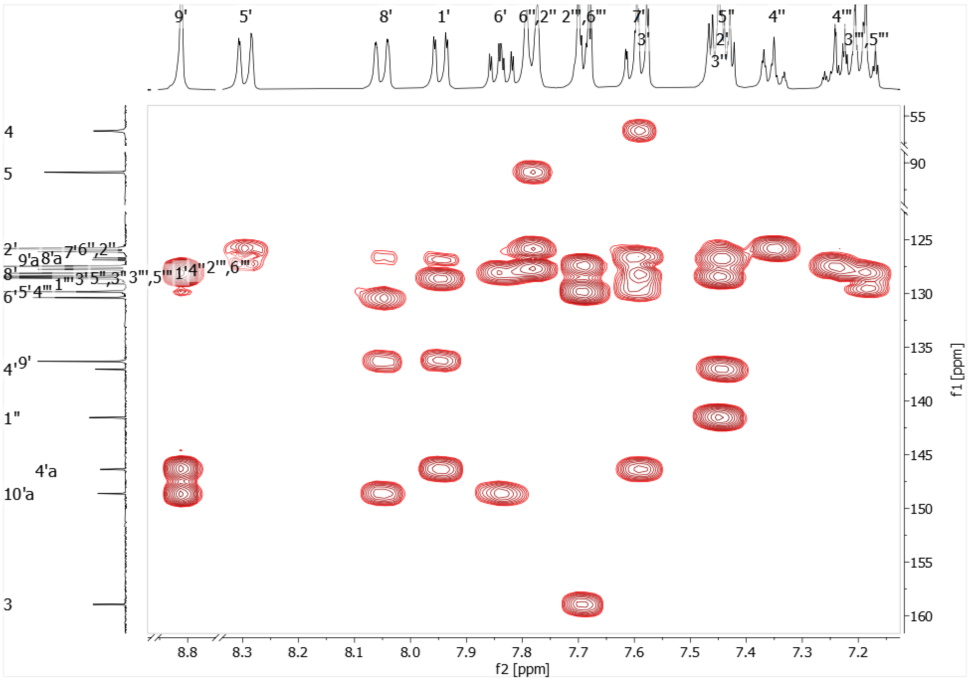

Figure S25. ^{1}H,^{13}C-HMBC ($CDCl_3$) NMR spectrum of derivative **7b**.

1.7 4-[3-(3-Nitrophenyl)-5-phenyl-4,5-dihydro-1,2-oxazol-4-yl]acridine (7d) and 4-[3-(3-nitrophenyl)-4-phenyl-4,5-dihydro-1,2-oxazole-5-yl]acridine (8d)

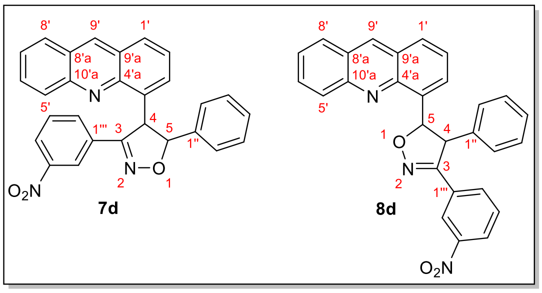

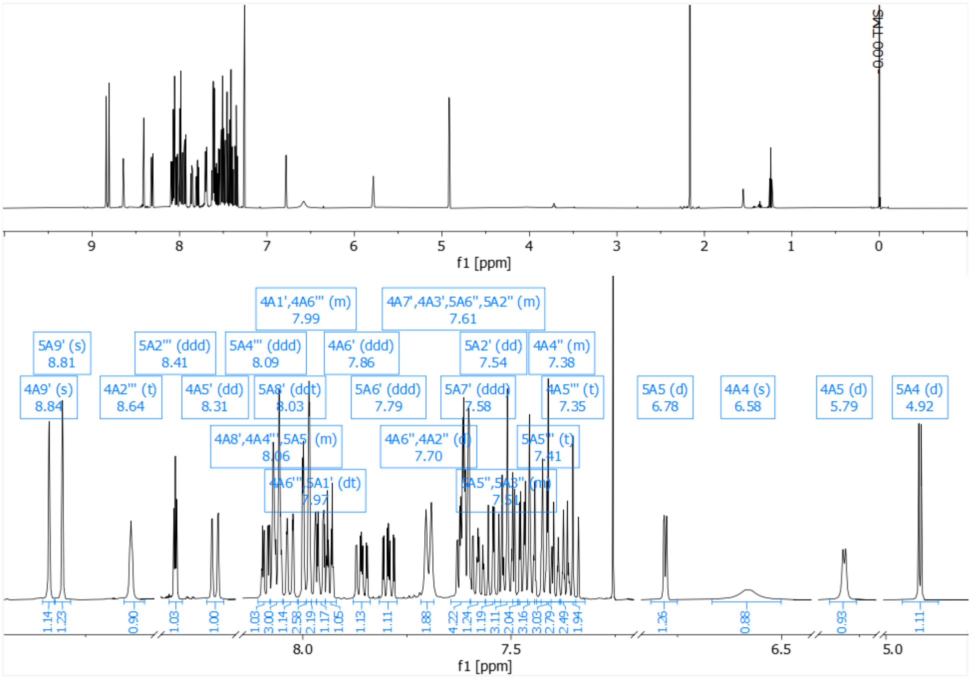

Figure S26. ^{1}H (600 MHz, $CDCl_3$) NMR spectrum of derivatives **7d** and **8d**.

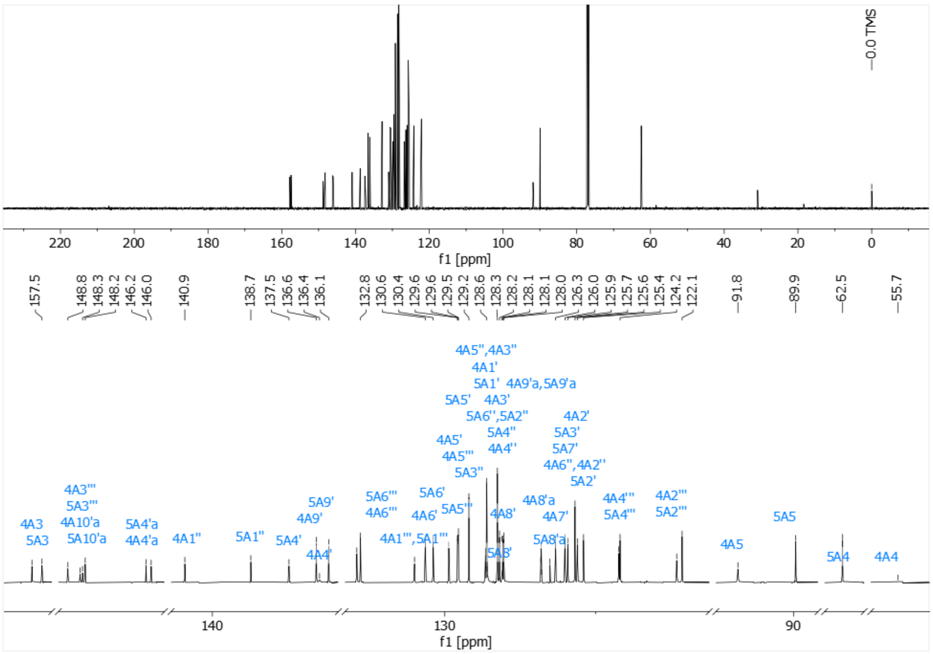

Figure S27. ^{13}C (150 MHz, $CDCl_3$) NMR spectrum of derivatives **7d** and **8d**.

1.8 4-[3-(4-Nitrophenyl)-5-phenyl-4,5-dihydro-1,2-oxazol-4-yl]acridine (7e) and 4-[3-(4-nitrophenyl)-4-phenyl-4,5-dihydro-1,2-oxazol-5-yl]acridine (8e)

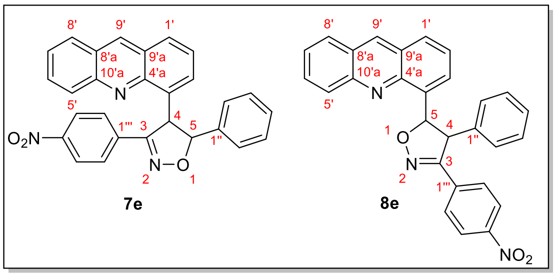

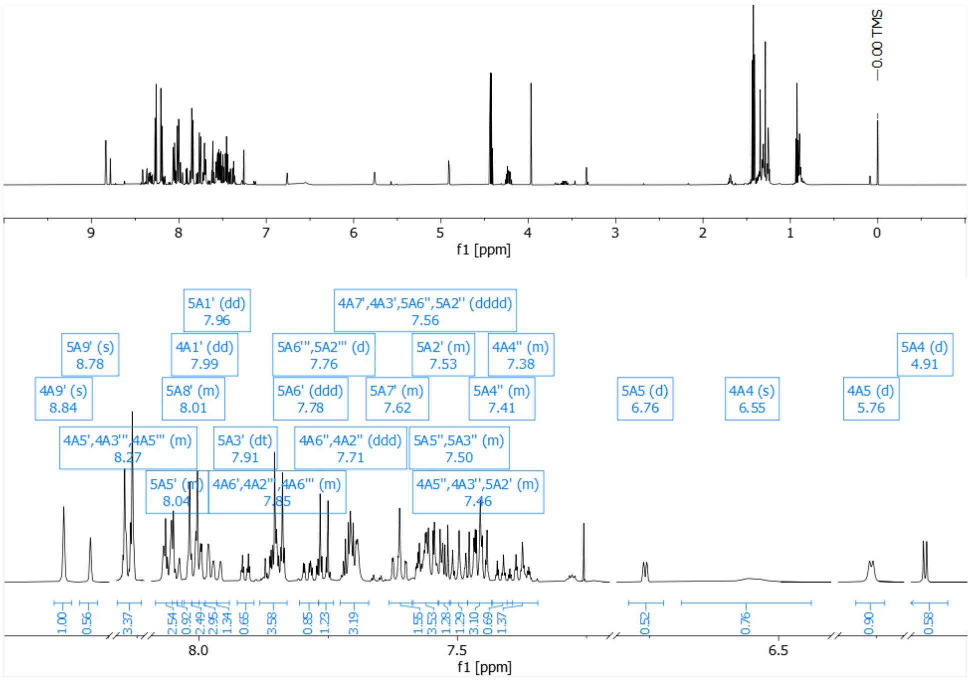

Figure S28. ^{1}H (600 MHz, $CDCl_3$) NMR spectrum of derivatives **7e** and **8e**.

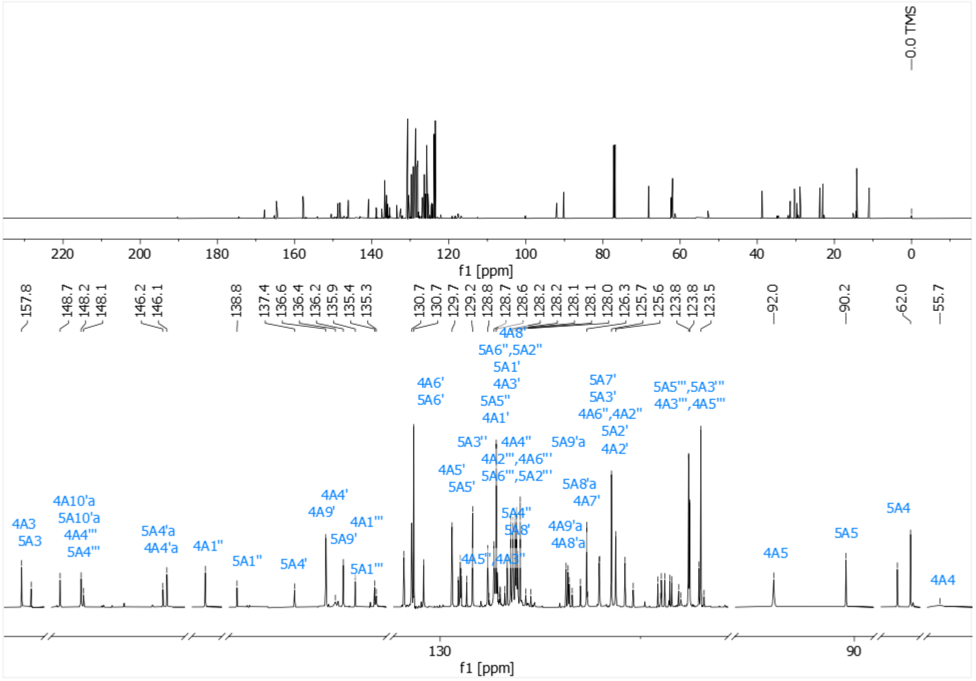

Figure S29. ^{13}C (150 MHz, $CDCl_3$) NMR spectrum of derivatives **7e** and **8e**.

1.9 4-[3-(4-Methoxyphenyl)-4-phenyl-4,5-dihydro-1,2-oxazol-5-yl]acridine (8a)

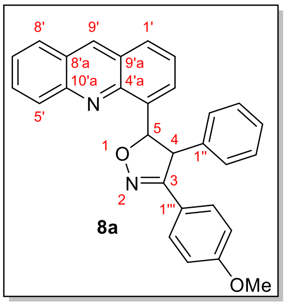

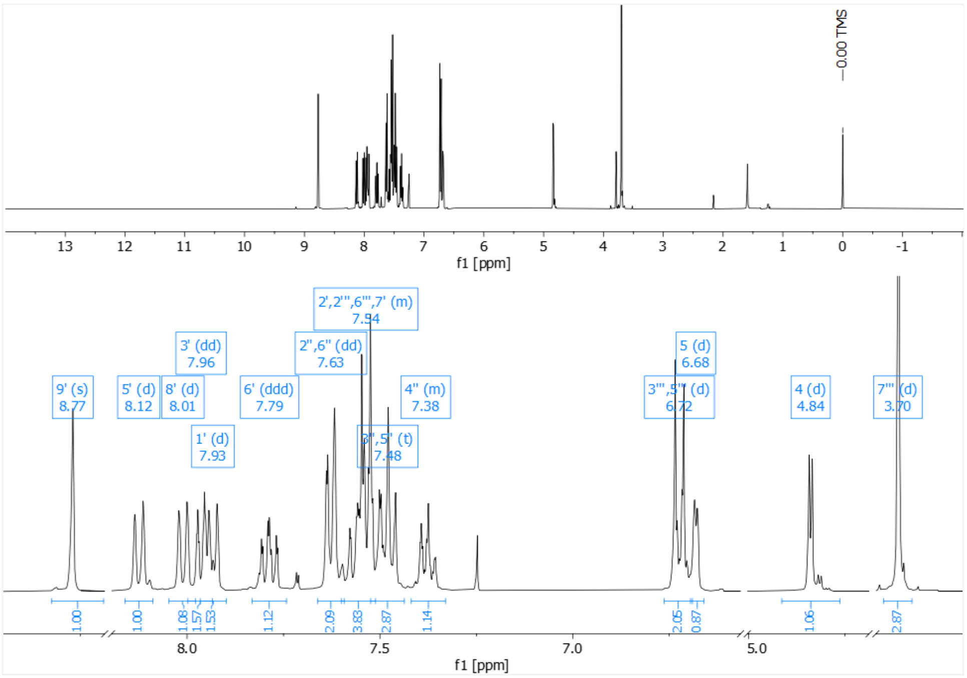

Figure S30. ¹H (400 MHz, CDCl₃) NMR spectrum of derivative **8a**.

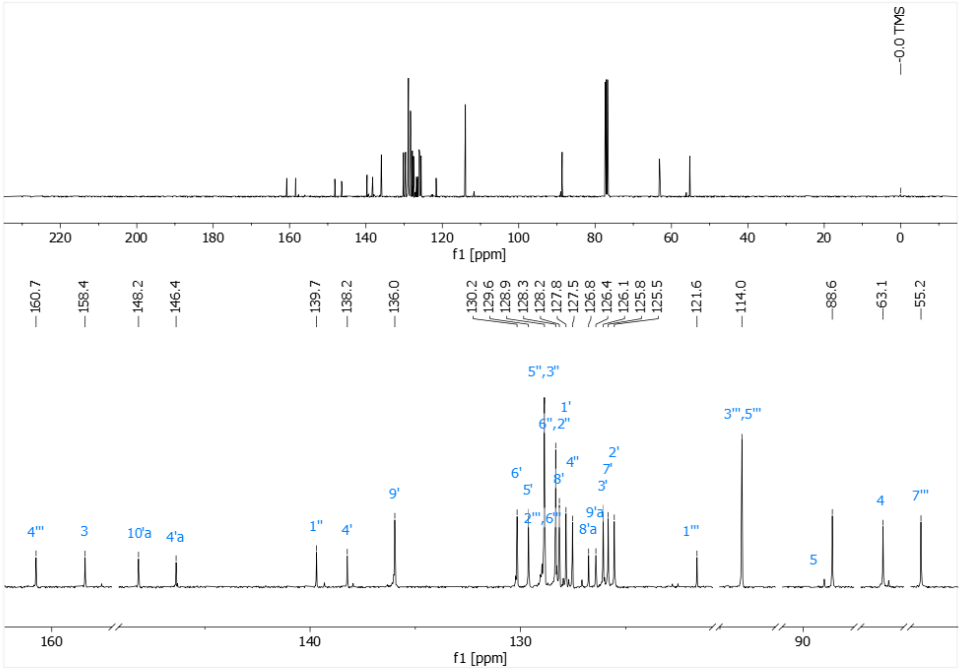

Figure S31. ¹³C (100 MHz, CDCl₃) NMR spectrum of derivative **8a**.

1.10 4-(3,4-Diphenyl-4,5-dihydro-1,2-oxazol-5-yl)acridine (8b)

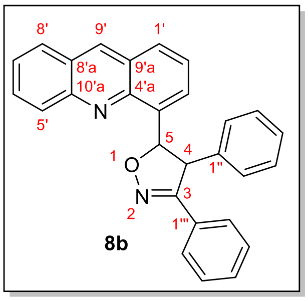

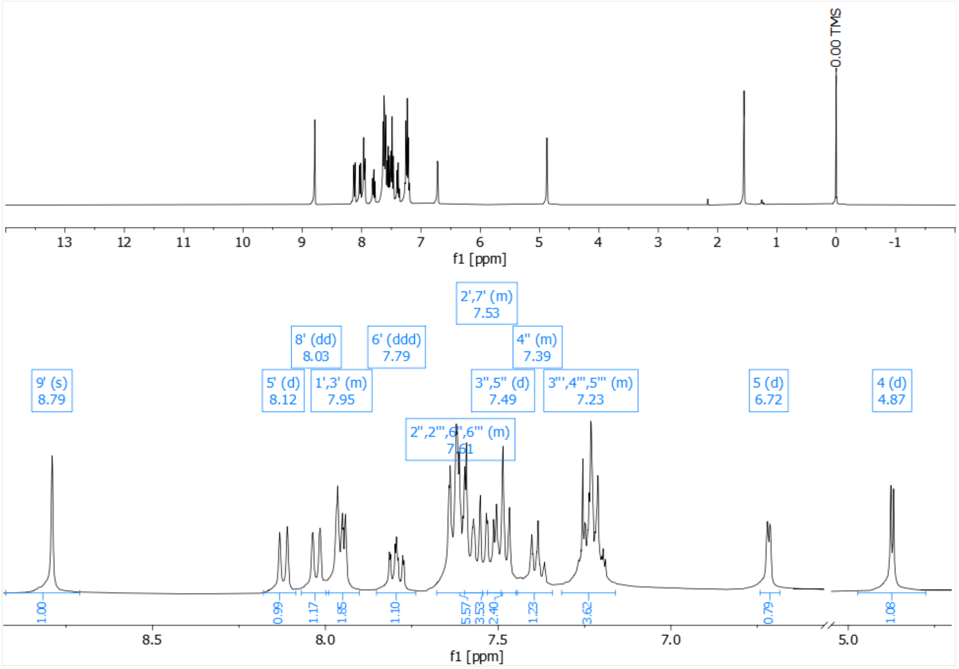

Figure S32. ^{1}H (400 MHz, $CDCl_3$) NMR spectrum of derivative **8b**.

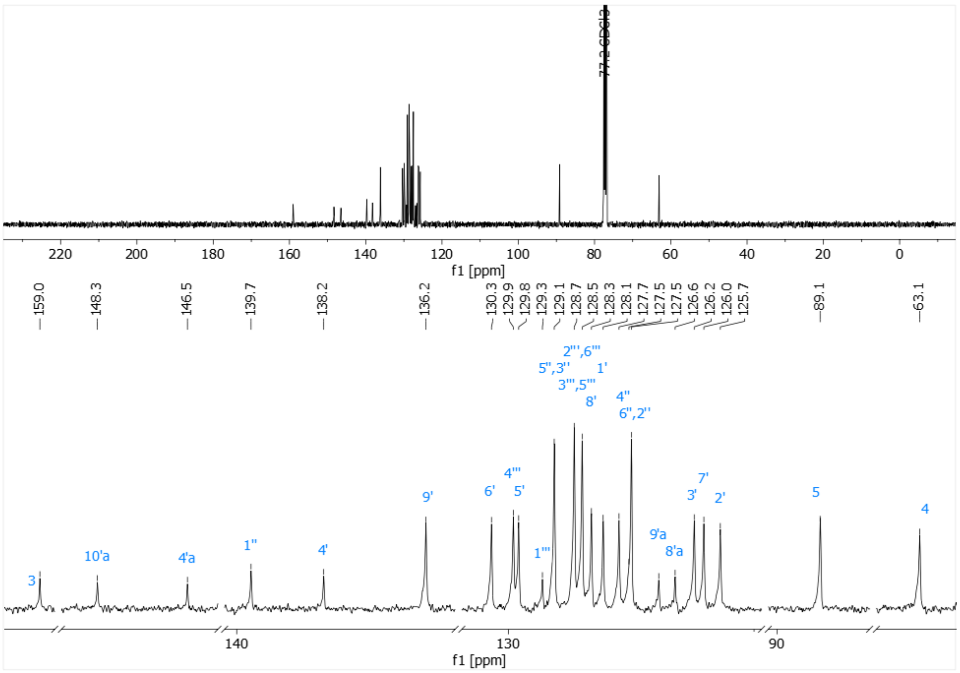

Figure S33. ^{13}C (100 MHz, $CDCl_3$) NMR spectrum of derivative **8b**.

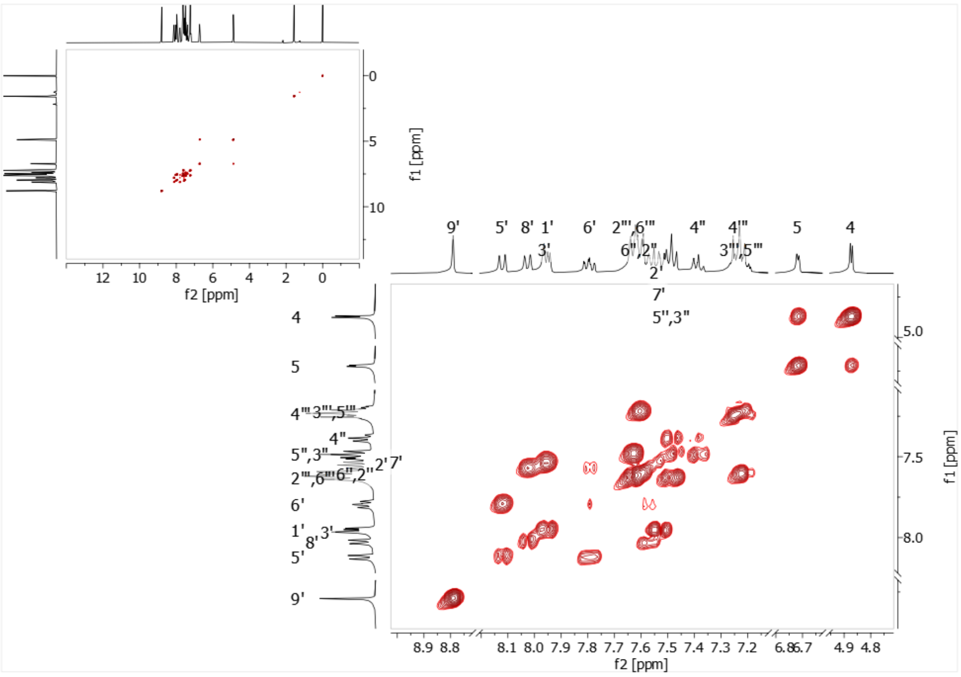

Figure S34. COSY ($CDCl_3$) NMR spectrum of derivative **8b**.

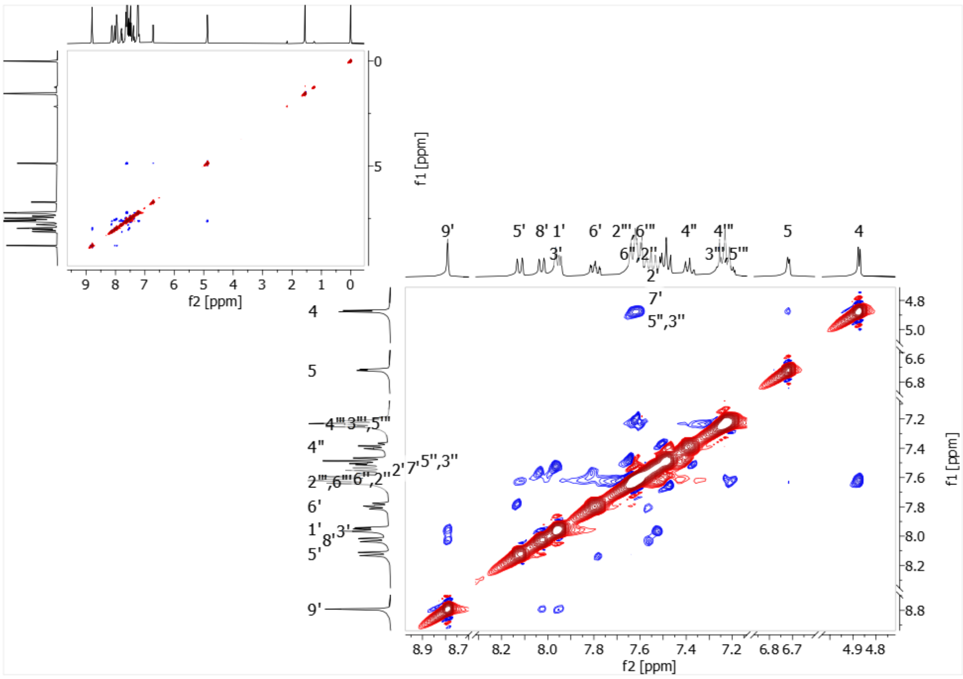

Figure S35. NOESY ($CDCl_3$) NMR spectrum of derivative **8b**.

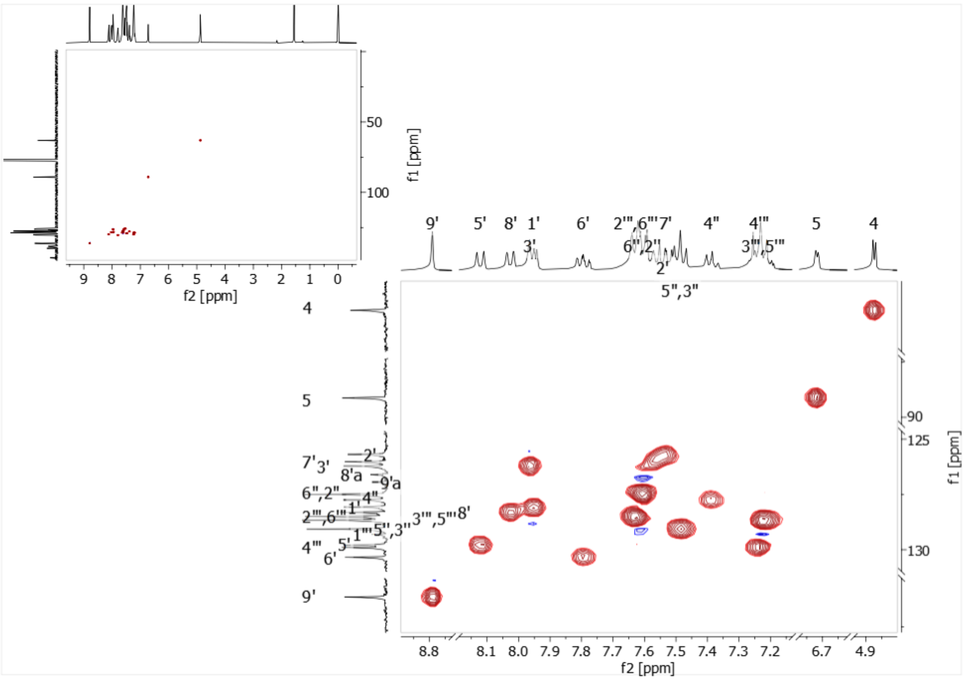

Figure S36. $^{1}H,^{13}C$-HSQC ($CDCl_3$) NMR spectrum of derivative **8b**.

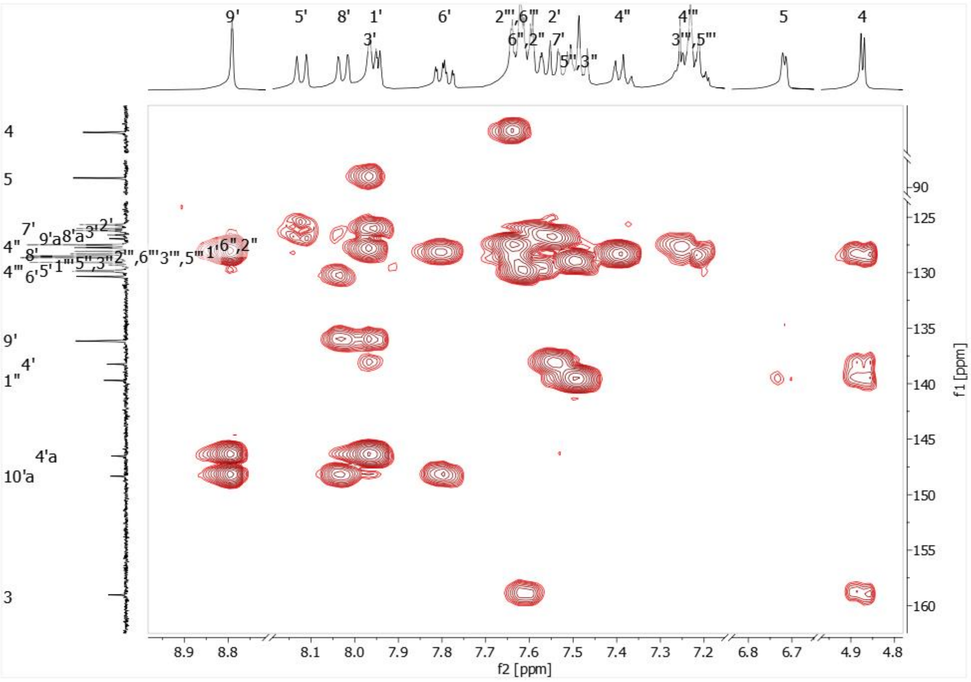

Figure S37. $^{1}H,^{13}C$-HMBC ($CDCl_3$) NMR spectrum of derivative **8b**.

1.11 4-[3-(4-Bromophenyl)-4-phenyl-4,5-dihydro-1,2-oxazol-5-yl]acridine (8c)

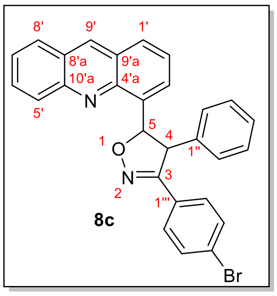

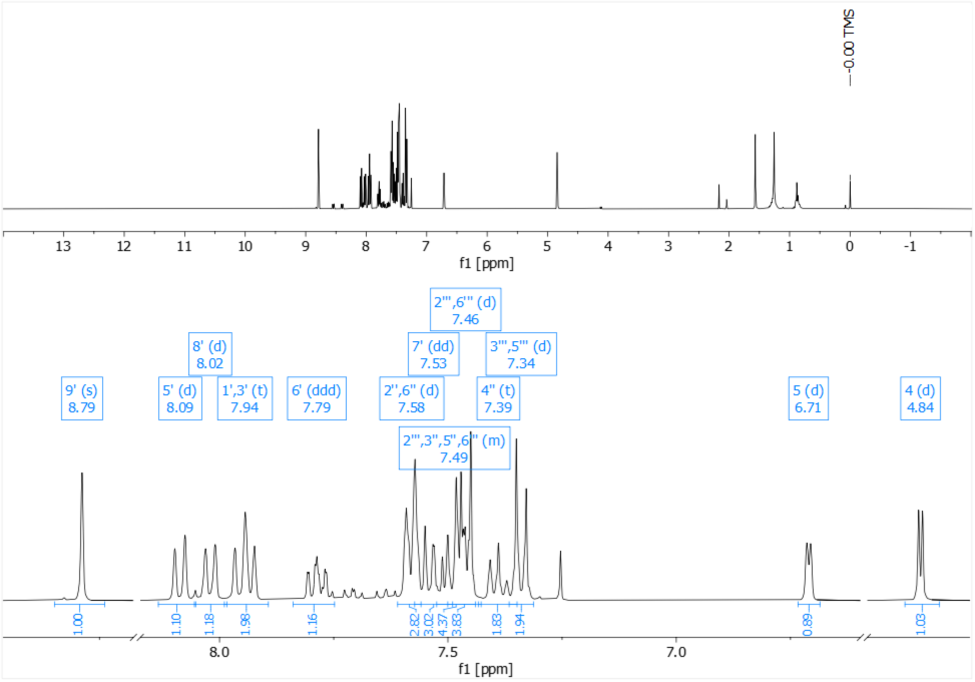

Figure S38. ¹H (400 MHz, $CDCl_3$) NMR spectrum of derivative **8c**.

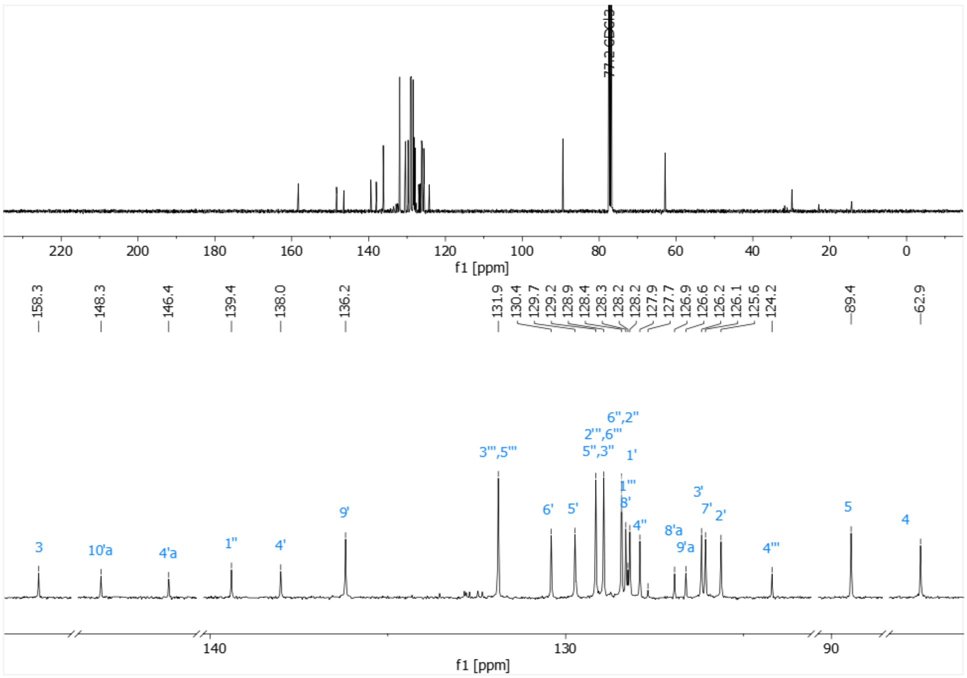

Figure S39. ¹³C (100 MHz, $CDCl_3$) NMR spectrum of derivative **8c**.

1.12 5-(Acridin-4-yl)-3-(4-methoxyphenyl)-4,5-dihydro-1,2-oxazole-4-carboxylic acid (9a)

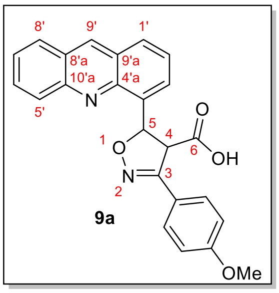

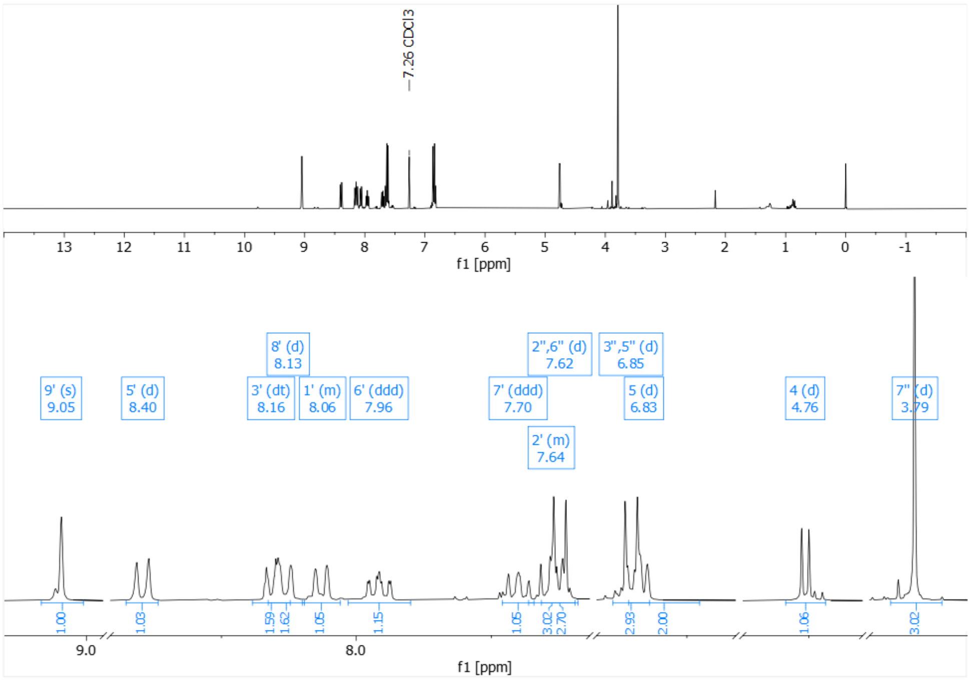

Figure S40. ^{1}H (400 MHz, $CDCl_3$) NMR spectrum of derivative **9a**.

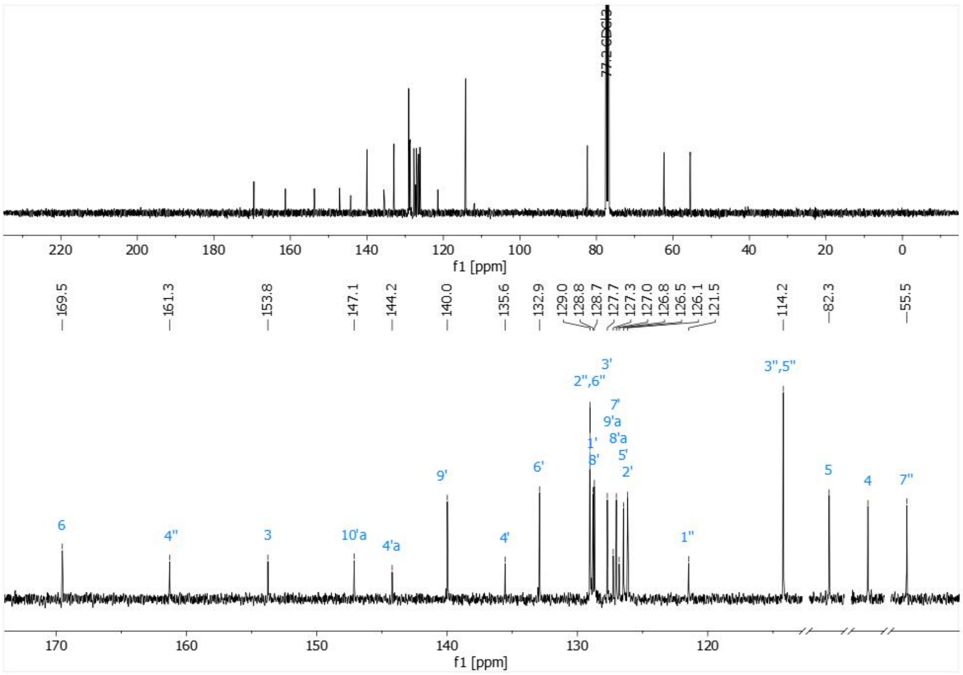

Figure S41. ^{13}C (100 MHz, $CDCl_3$) NMR spectrum of derivative **9a**.

1.13 5-(Acridin-4-yl)-3-phenyl-4,5-dihydro-1,2-oxazole-4-carboxylic acid (9b)

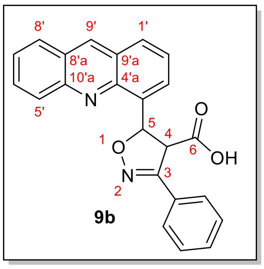

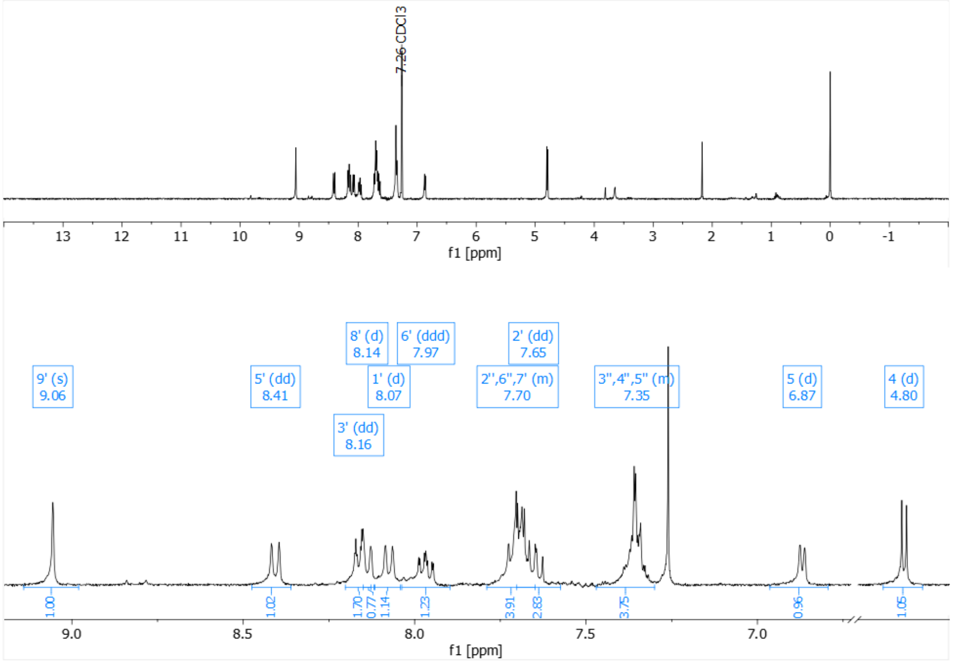

Figure S42. ^{1}H (400 MHz, $CDCl_3$) NMR spectrum of derivative **9b**.

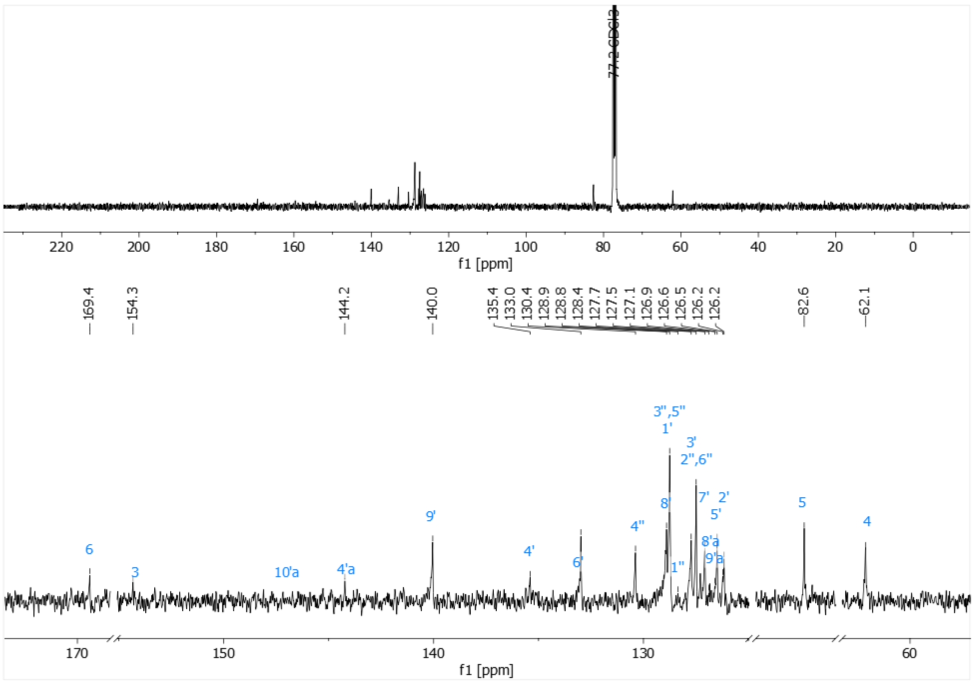

Figure S43. ^{13}C (100 MHz, $CDCl_3$) NMR spectrum of derivative **9b**.

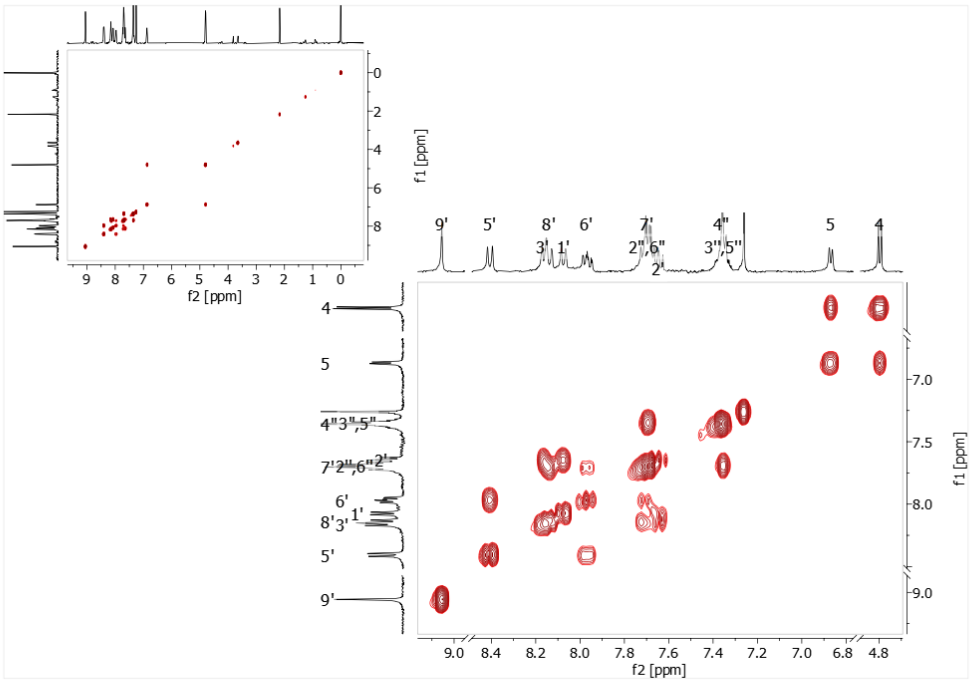

Figure S44. COSY ($CDCl_3$) NMR spectrum of derivative **9b**.

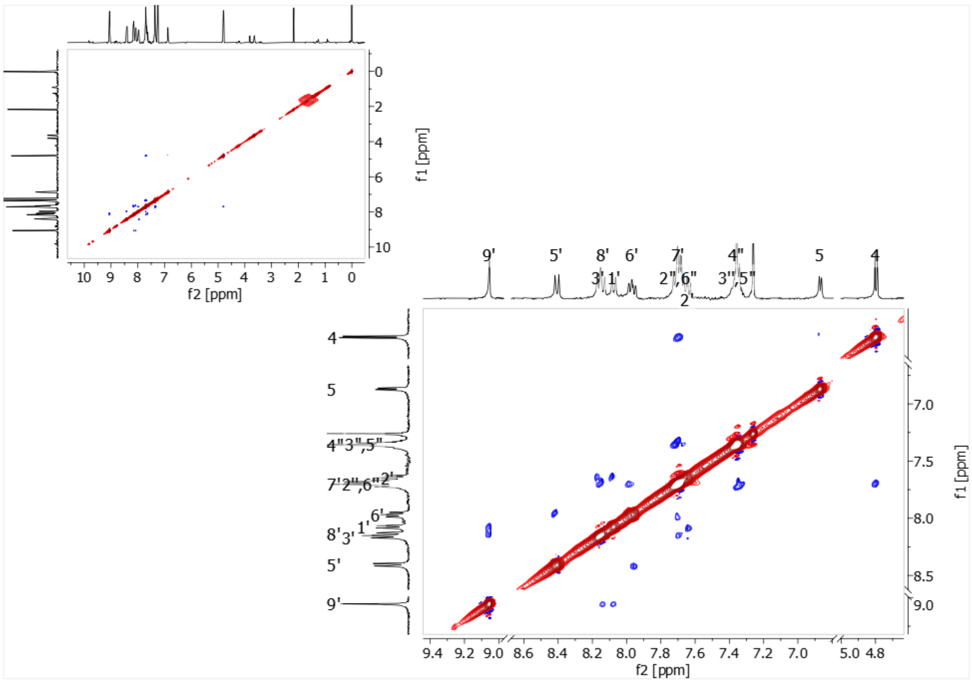

Figure S45. NOESY ($CDCl_3$) NMR spectrum of derivative **9b**.

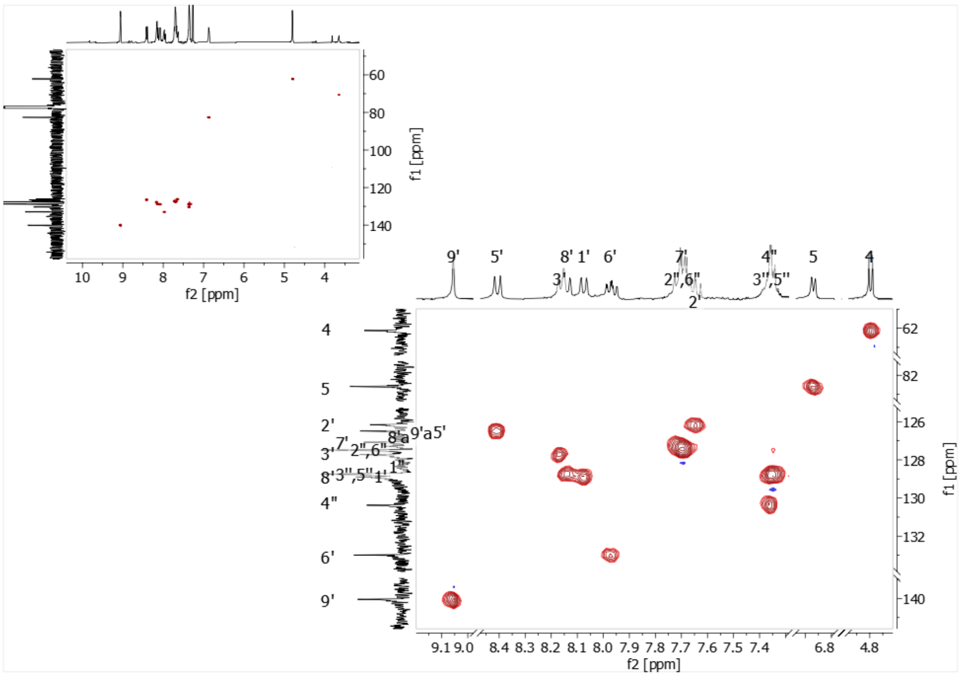

Figure S46. $^{1}H,^{13}C$-HSQC ($CDCl_3$) NMR spectrum of derivative **9b**.

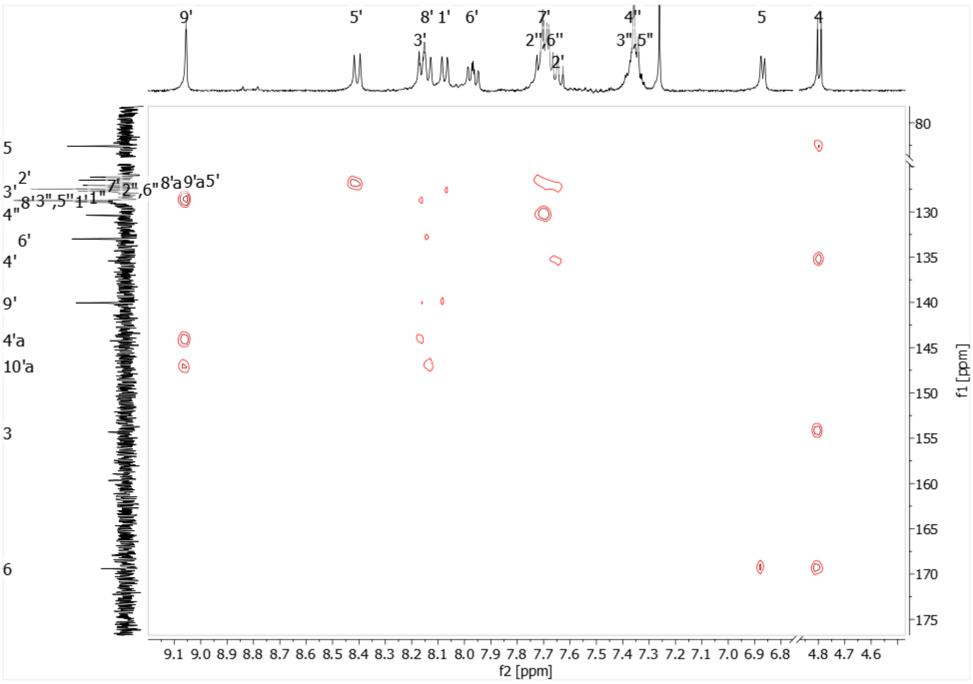

Figure S47. $^{1}H,^{13}C$-HMBC ($CDCl_3$) NMR spectrum of derivative **9b**.

1.14 5-(Acridin-4-yl)-3-(3-nitrophenyl)-4,5-dihydro-1,2-oxazole-4-carboxylic acid (9d) and 4-[3-(3-nitrophenyl)-4,5-dihydro-1,2-oxazol-5-yl]acridine (11d)

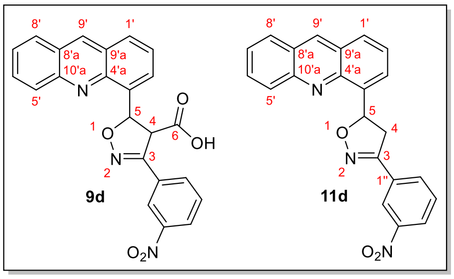

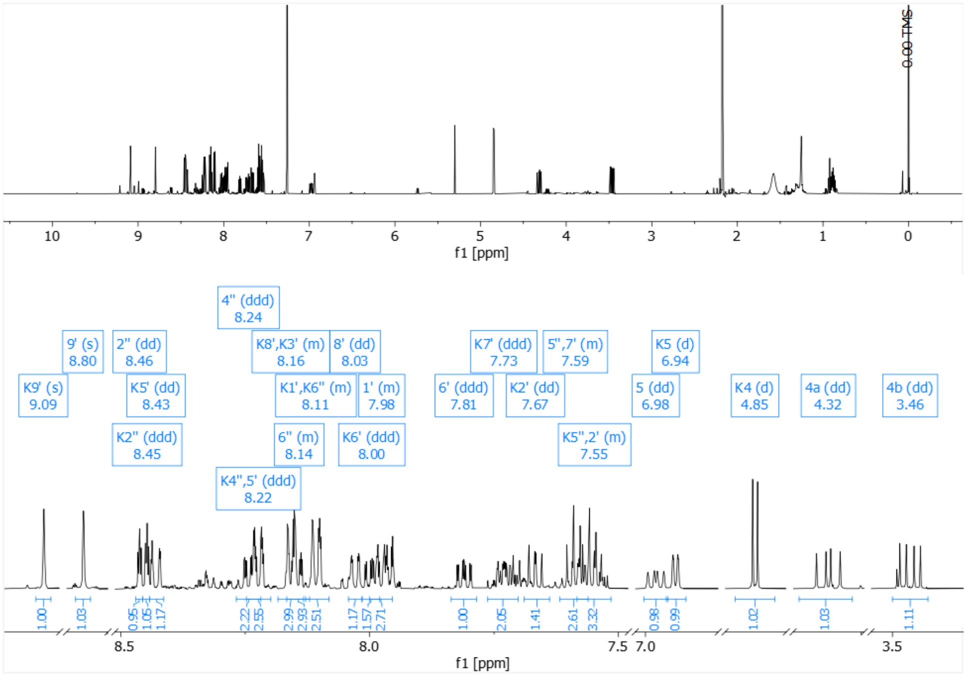

Figure S48. ^{1}H (400 MHz, $CDCl_3$) NMR spectrum of derivatives **9d** and **11d**.

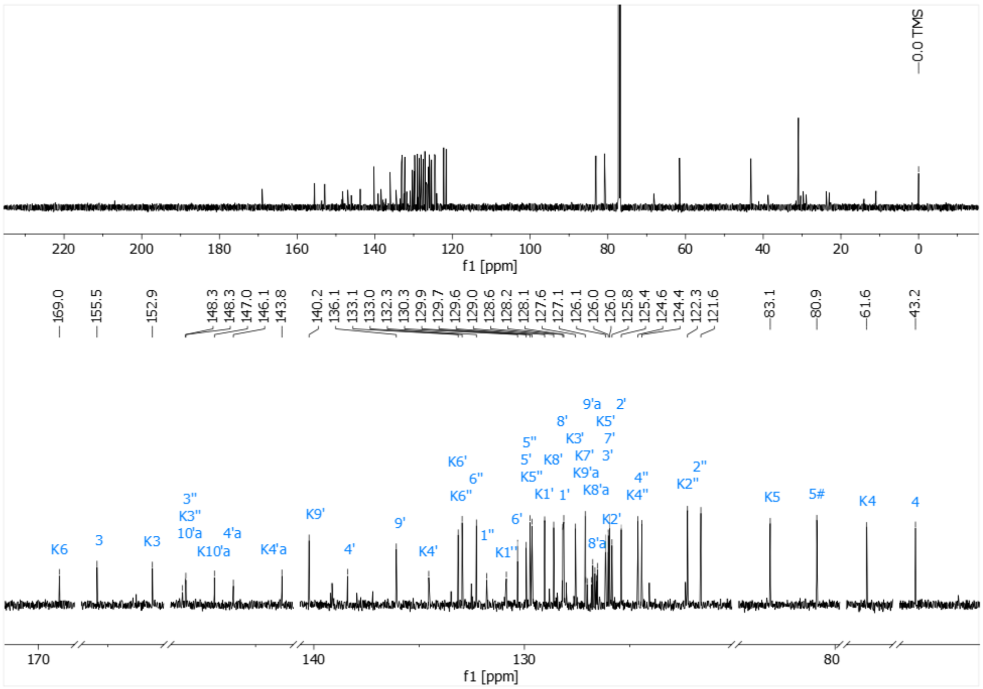

Figure S49. ^{13}C (100 MHz, $CDCl_3$) NMR spectrum of derivatives **9d** and **11d**.

1.15 5-(Acridin-4-yl)-3-(4-nitrophenyl)-4,5-dihydro-1,2-oxazole-4-carboxylic acid (9e)

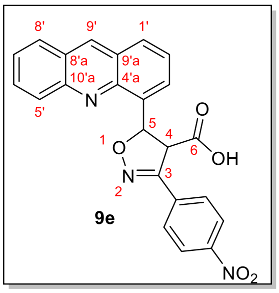

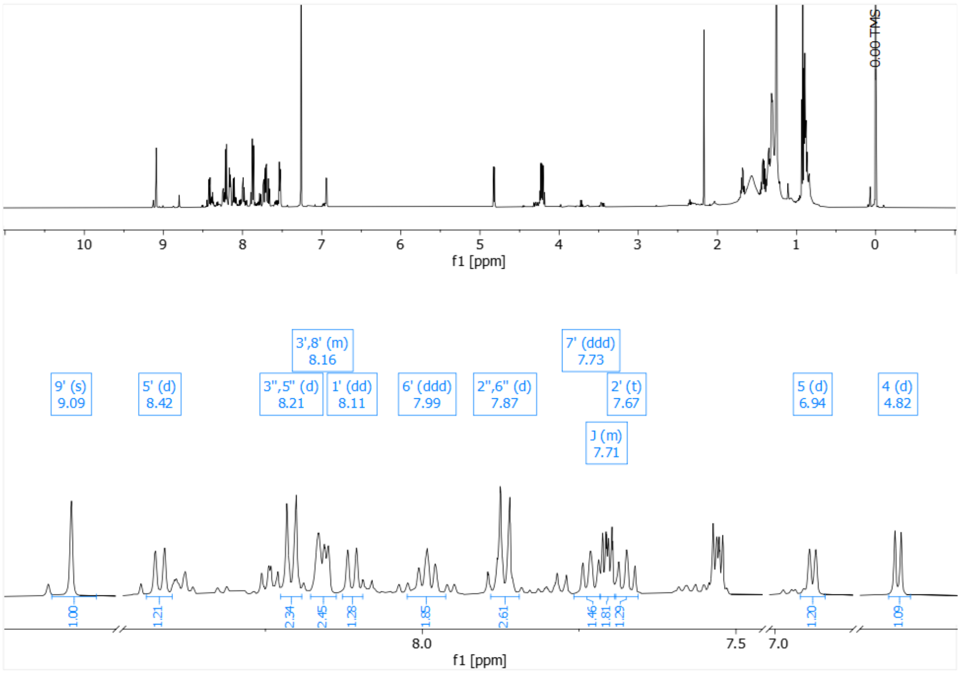

Figure S50. ^{1}H (400 MHz, $CDCl_3$) NMR spectrum of derivative **9e**.

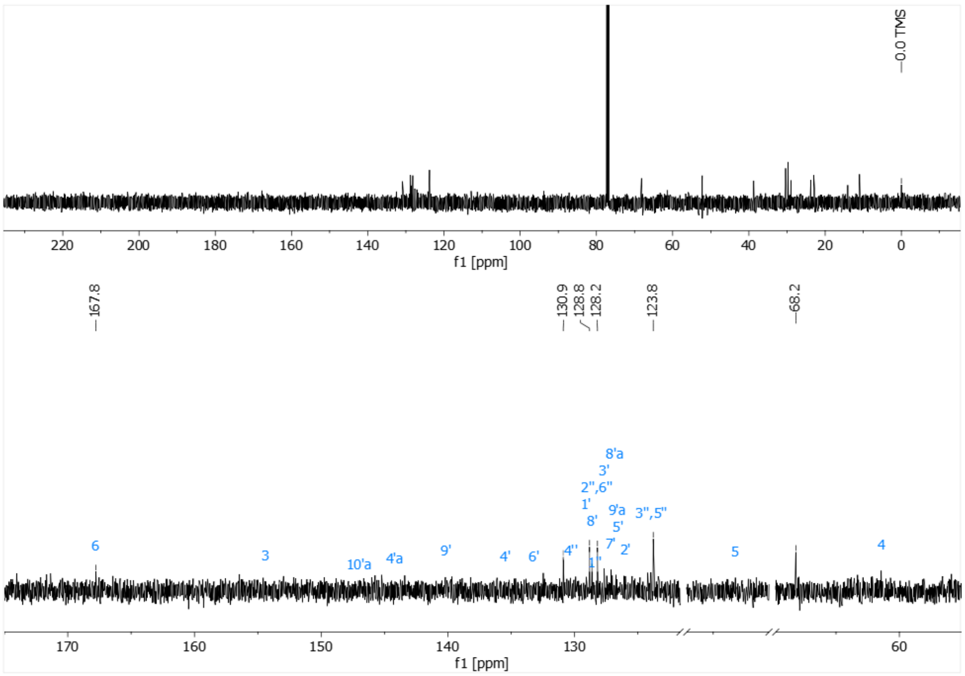

Figure S51. ^{13}C (100 MHz, $CDCl_3$) NMR spectrum of derivative **9e**.

1.16 (4*Z*)-4-[(Acridin-4-yl)methylidene)]-3-(4-nitrophenyl)-4,5-dihydro-1,2-oxazol-5-one (*Z*-10e)

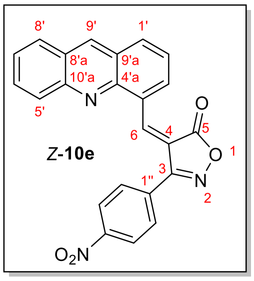

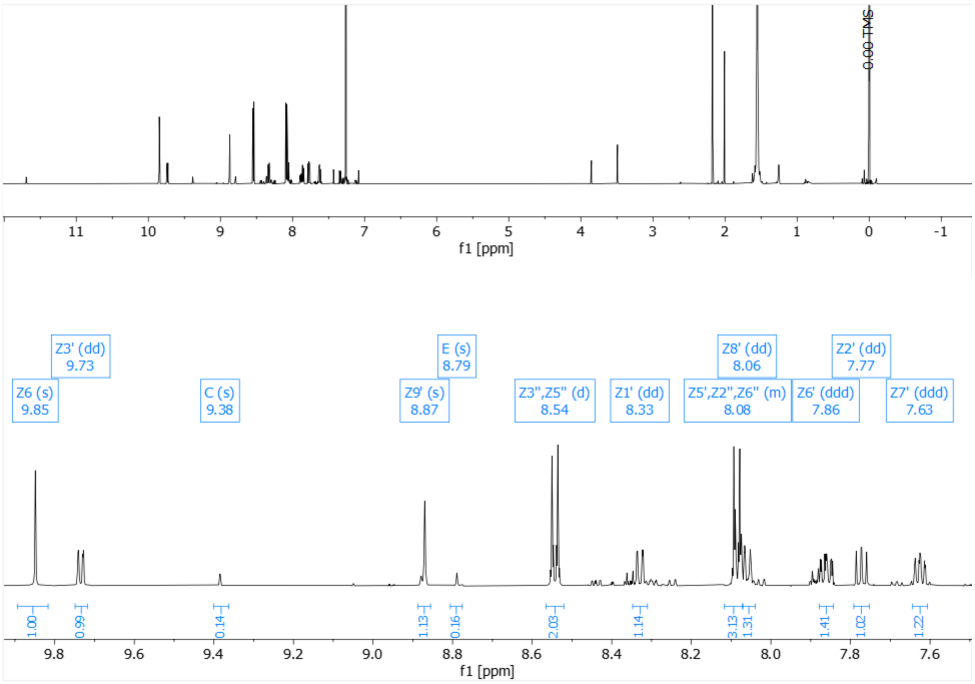

Figure S52. ^{1}H (600 MHz, CDCl3) NMR spectrum of derivative *Z*-**10e**.

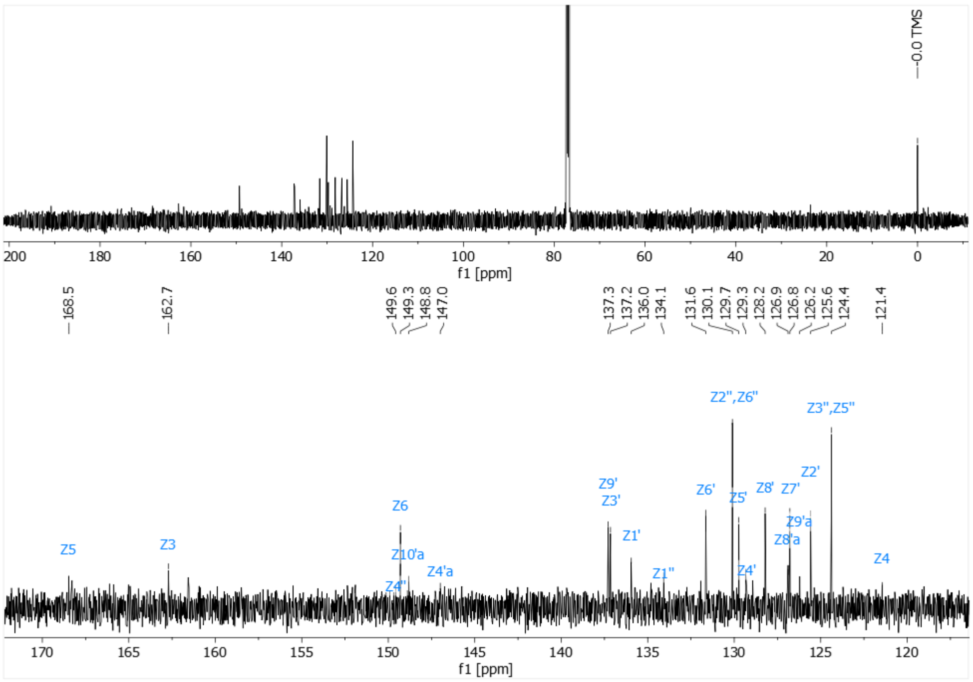

Figure S53. ^{13}C (150 MHz, CDCl3) NMR spectrum of derivative *Z*-**10e**.

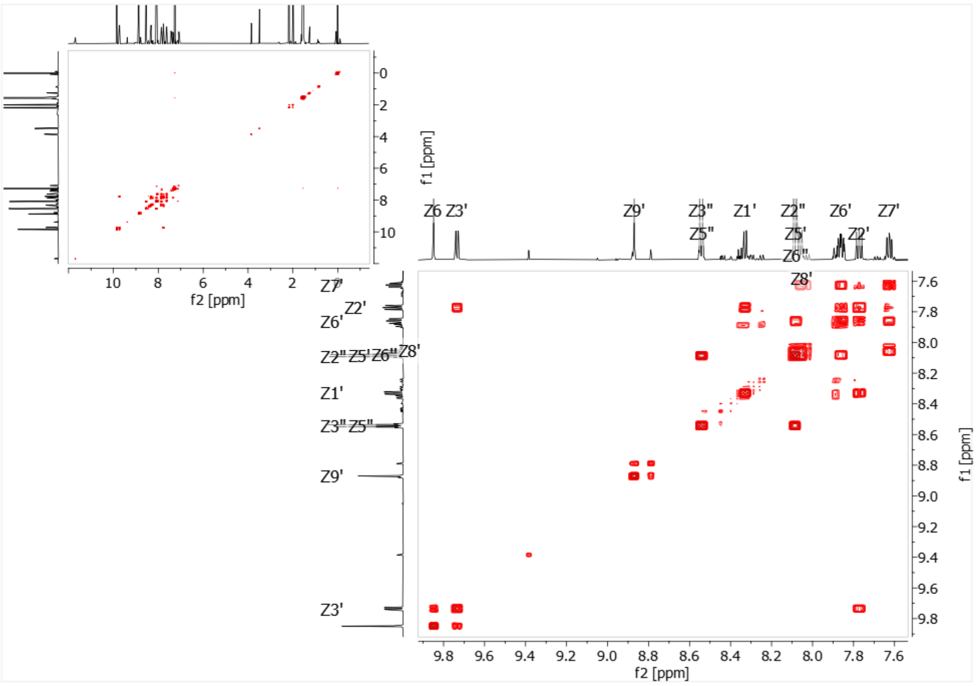

Figure S54. COSY ($CDCl_3$) NMR spectrum of derivative *Z*-**10e**.

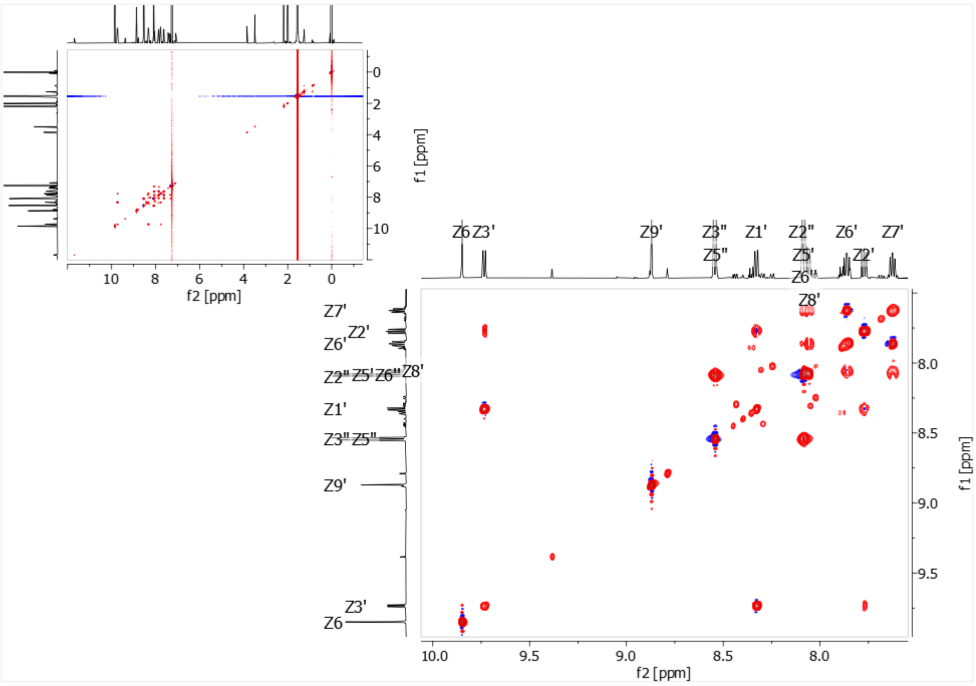

Figure S55. TOCSY ($CDCl_3$) NMR spectrum of derivative *Z*-**10e**.

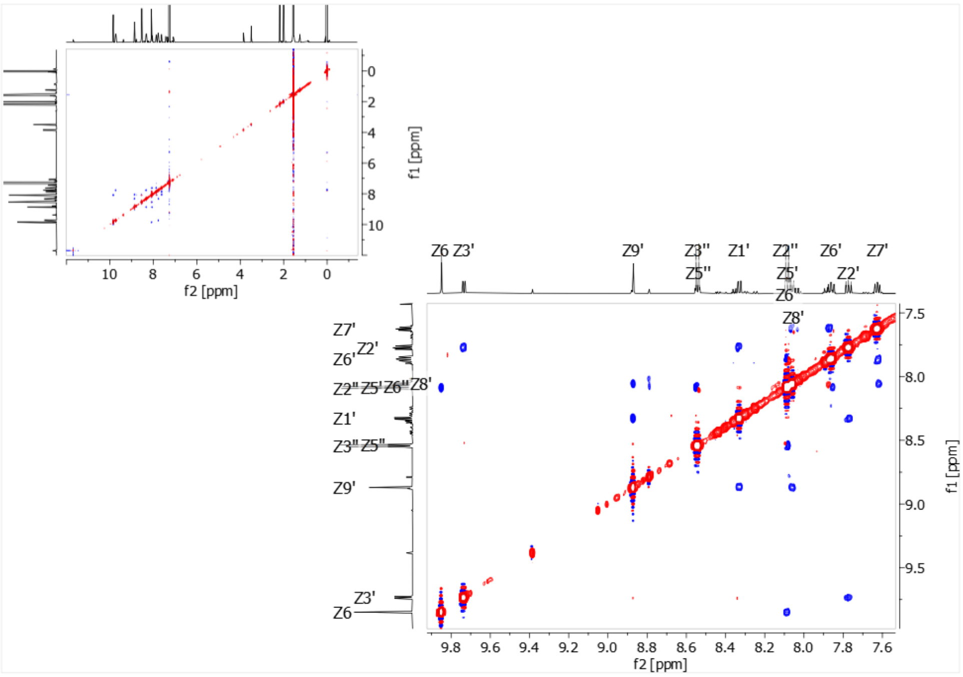

Figure S56. NOESY ($CDCl_3$) NMR spectrum of derivative *Z*-**10e**.

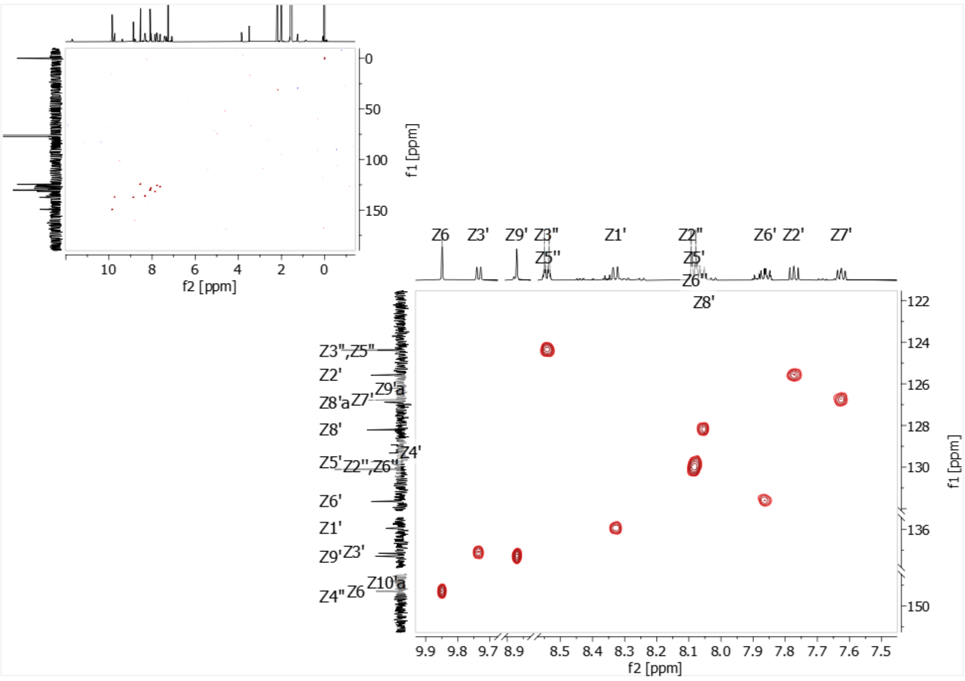

Figure S57. $^1H,^{13}C$-HSQC ($CDCl_3$) NMR spectrum of derivative *Z*-**10e**.

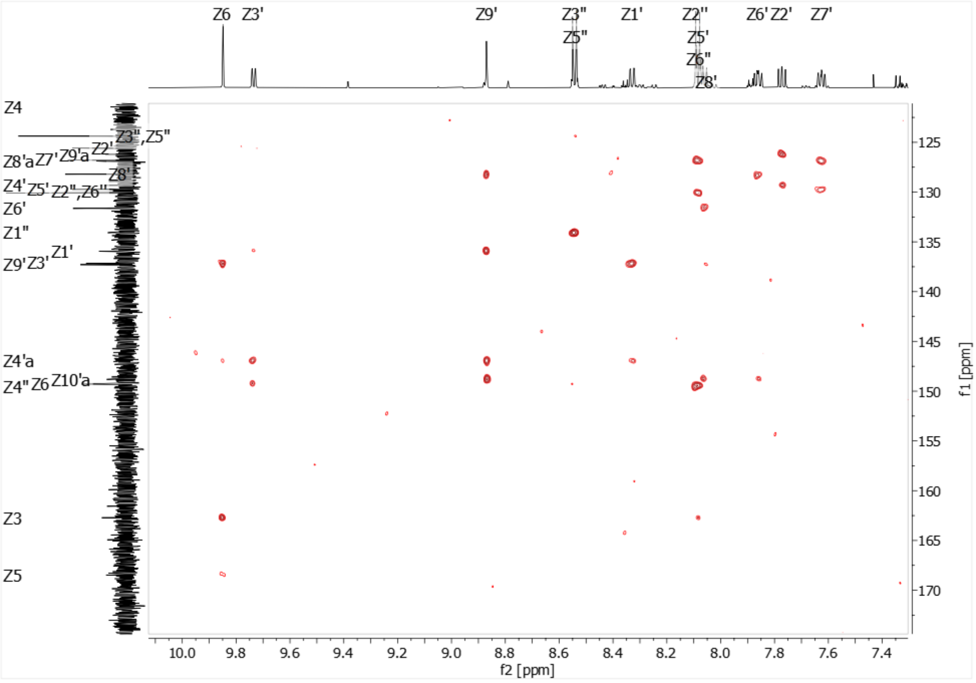

Figure S58. ^{1}H,^{13}C-HSQC ($CDCl_3$) NMR spectrum of derivative *Z*-**10e**.

1.17 4-[3-(4-Methoxyphenyl)-4,5-dihydro-1,2-oxazol-5-yl]acridine (11a)

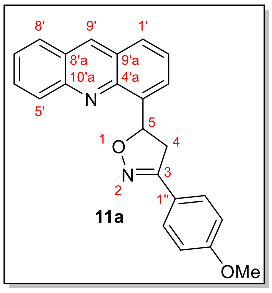

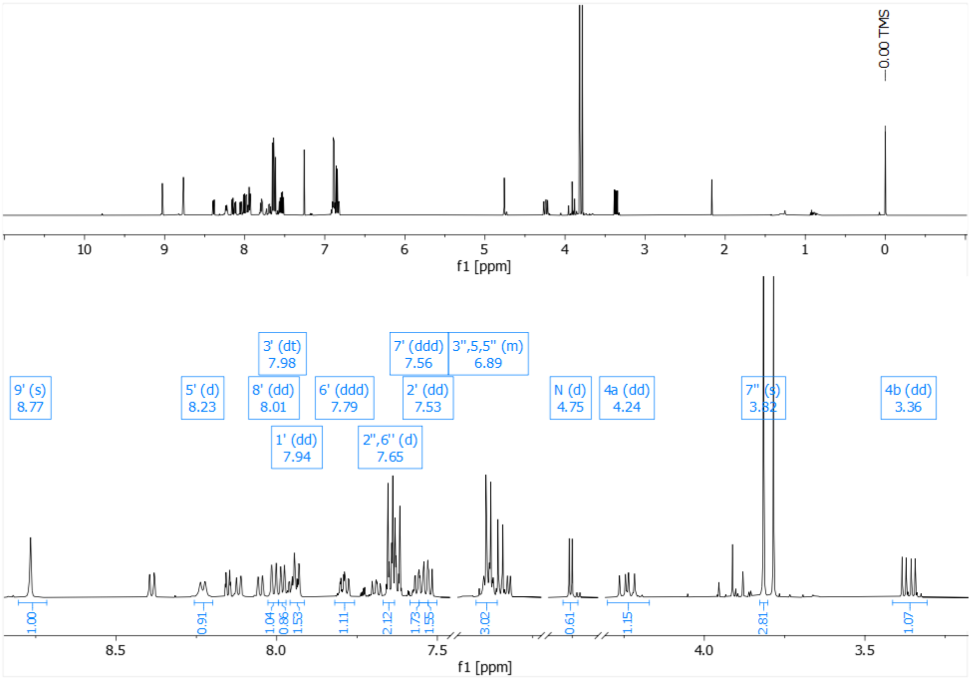

Figure S59. ^{1}H (600 MHz, CDCl3) NMR spectrum of derivative **11a**.

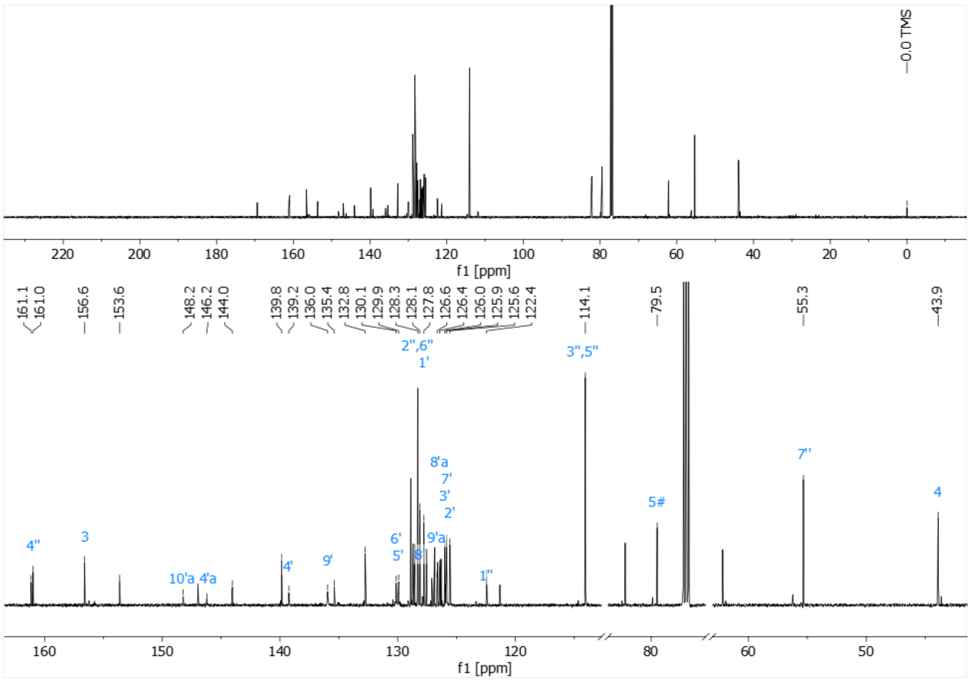

Figure S60. ^{13}C (150 MHz, CDCl3) NMR spectrum of derivative **11a**.

1.18 4-(3-Phenyl-4,5-dihydro-1,2-oxazol-5-yl)acridine (11b)

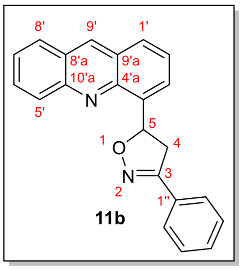

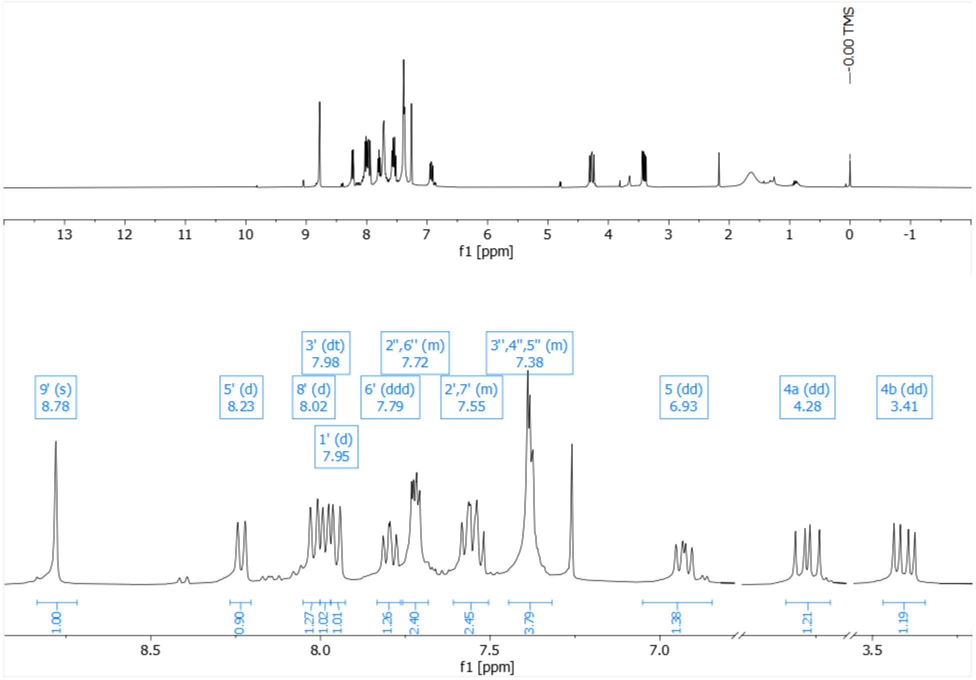

Figure S61. ^{1}H (400 MHz, $CDCl_3$) NMR spectrum of derivative **11b**.

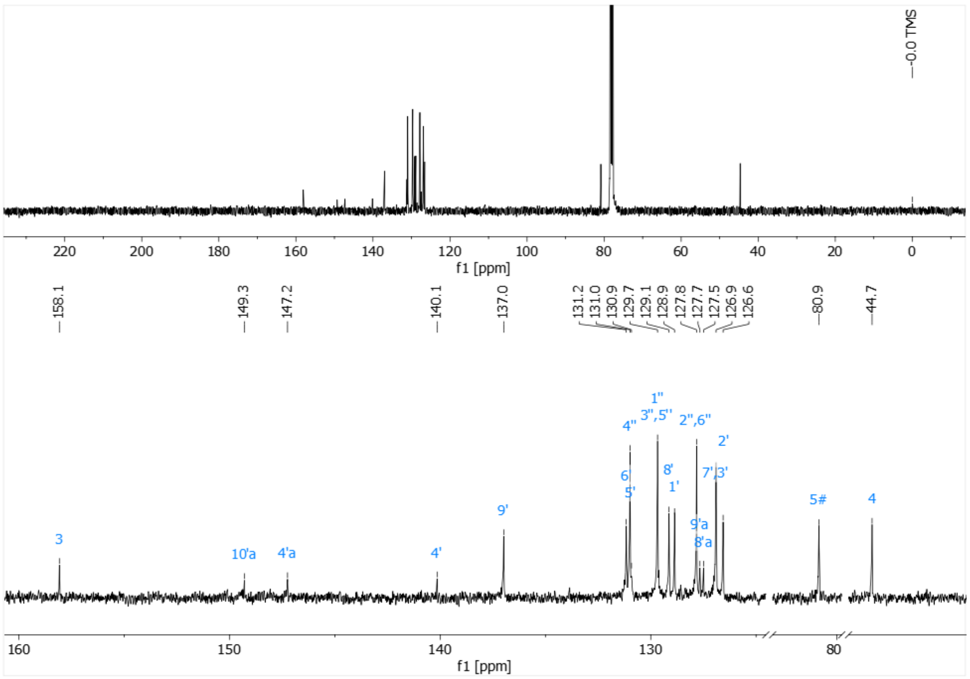

Figure S62. ^{13}C (100 MHz, $CDCl_3$) NMR spectrum of derivative **11b**.

1.19 4-[3-(4-Nitrophenyl)-4,5-dihydro-1,2-oxazol-5-yl]acridine (11e)

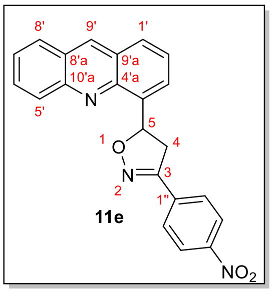

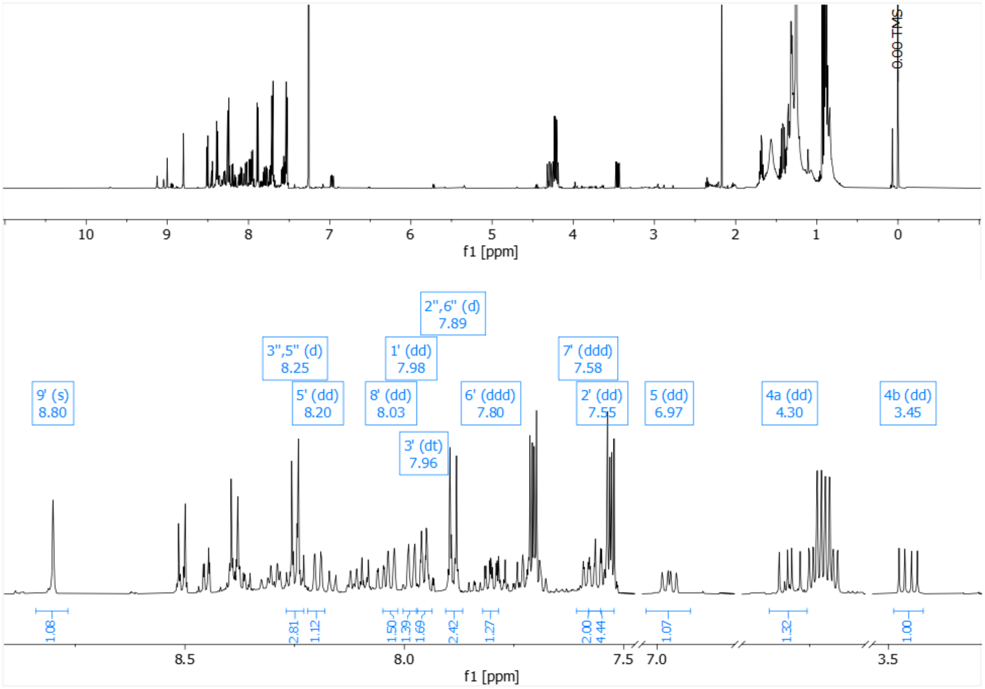

Figure S63. ^{1}H (600 MHz, $CDCl_3$) NMR spectrum of derivative **11e**.

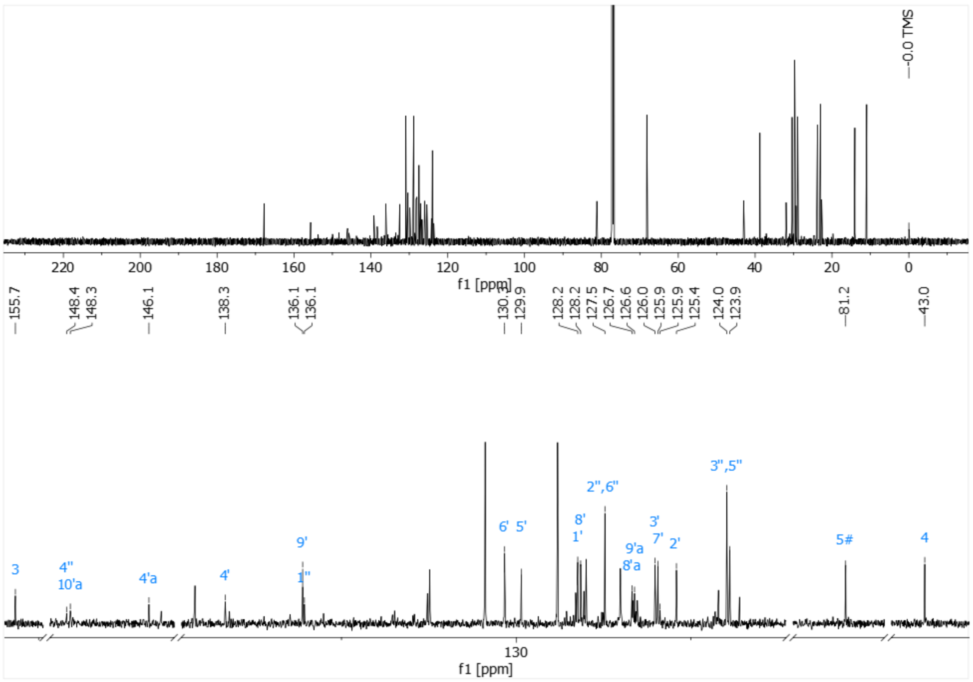

Figure S64. ^{13}C (150 MHz, $CDCl_3$) NMR spectrum of derivative **11e**.
